# Dynamic Co‐Evolution of Obesity‐Metabolic‐Inflammatory for Cardiovascular Disease Risk Stratification in Middle‐Aged and Older Adults: A Data‐Driven Joint Trajectory Analysis

**DOI:** 10.1002/clc.70429

**Published:** 2026-07-29

**Authors:** Qinggao Wang, Yilu Lei, Qingfeng Zhou, Yan Long, Yinjuan Lai

**Affiliations:** ^1^ Department of Cardiology The First Affiliated Hospital of Guangxi University of Chinese Medicine Nanning China; ^2^ Geriatric Ward The First Affiliated Hospital of Guangxi University of Chinese Medicine Nanning China; ^3^ Department of Scientific Research The First Affiliated Hospital of Guangxi University of Chinese Medicine Nanning China; ^4^ Department of Hospital Infection Management The First Affiliated Hospital of Guangxi University of Chinese Medicine Nanning China

**Keywords:** body mass index, cardiovascular disease, cluster analysis, C‐reactive protein, joint trajectories, triglyceride‐glucose index

## Abstract

**Background:**

Cardiovascular disease (CVD) risk assessment based on single‐time‐point measurements may inadequately capture the dynamic interplay of obesity, metabolic dysfunction, and chronic inflammation. Understanding their long‐term co‐evolution may improve CVD risk stratification.

**Methods:**

We conducted a prospective cohort study using data from the China Health and Retirement Longitudinal Study. Participants aged ≥ 45 years with repeated measurements of body mass index (BMI), triglyceride‐glucose (TyG) index, and high‐sensitivity C‐reactive protein (hsCRP) between 2011 and 2015 were included. Joint trajectories of these obesity‐metabolic‐inflammatory markers were identified using the KmL3D clustering algorithm. Incident CVD (heart disease or stroke) was ascertained during follow‐up from 2015 to 2020. Cox proportional hazards models were applied to evaluate associations between trajectory groups and CVD risk after adjustment for demographic, lifestyle, and clinical covariates.

**Results:**

Among 4483 participants (mean age 57.8 years; 52.6% women), four distinct joint trajectory patterns were identified: a sustained low‐risk profile (46.8%), an obesity‐predominant profile (26.7%), a metabolic–obesity‐driven profile (17.1%), and an inflammation‐dominant profile (9.4%). During a median follow‐up of 5.0 years, 592 incident CVD events occurred. Compared with the low‐risk profile, all adverse trajectory groups were associated with significantly higher CVD risk, with hazard ratios ranging from 1.35 to 1.46 after full adjustment. Associations were more pronounced for stroke, particularly among individuals with a metabolic–obesity‐driven trajectory. Findings were robust across sensitivity analyses.

**Conclusions:**

Distinct dynamic co‐evolution patterns of obesity, metabolic dysfunction, and inflammation identify population subgroups with substantially different risks of incident CVD.

## Graphical abstract

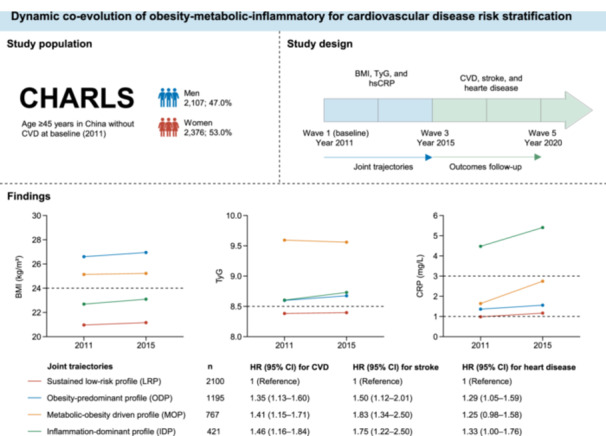

## 1 Introduction

Cardiovascular disease (CVD) is the leading cause of global mortality and disability [1]. A persistent clinical challenge is the significant variation in actual CVD outcomes among individuals with similar traditional risk scores [2, 3, 4], despite the widespread application of models like the ACC/AHA Pooled Cohort Equations (PCEs) and the European SCORE2 system [5, 6]. This discrepancy particularly underscores the limitations of current tools in adequately capturing risk across diverse and heterogeneous populations, where factors such as genetic background, socioeconomic context, and lifestyle may differentially influence disease progression [7, 8]. To address this, the field must increasingly integrate novel biomarkers, advanced imaging, and other personalised assessment methods. This evolution is crucial for developing more nuanced risk prediction strategies that can better account for individual variability and improve clinical decision‐making in cardiovascular prevention.

Recent research identifies metabolic dysregulation, chronic low‐grade inflammation, and obesity as interconnected drivers of atherosclerosis. The triglyceride‐glucose (TyG) index serves as a reliable marker of insulin resistance [9]; high‐sensitivity C‐reactive protein (hsCRP) is a standard indicator of systemic inflammation [10]; and body mass index (BMI) commonly reflects obesity [11]. Evidence shows that these factors not only independently correlate with CVD risk [12, 13, 14], but also interact synergistically [15, 16, 17, 18]. A nationwide prospective cohort study in China found that the TyG index mediated over 50% of the association between BMI and stroke; moreover, the combination of high BMI and high TyG was linked to a more than twofold increase in stroke risk [17]. Another study, combining TyG and hsCRP into a single metric, demonstrated superior predictive performance for three‐vessel coronary artery disease compared with either marker alone [16]. Collectively, these findings suggest that the interplay among these three domains—obesity, metabolism, and inflammation—is central to cardiovascular pathophysiology and that their joint dynamic patterns may carry more prognostic weight than any isolated measurement.

Recently, a conceptual shift has occurred in analytical approaches used to assess associations with CVD [19], moving away from exploring metabolic, inflammatory, and obesity as independent exposures toward using more advanced approaches to study the effects of these biomarkers with change over time on various health outcomes [19, 20]. Among newer analytical approaches, K‐means trajectory clustering and the latent class growth model have been most frequently used for studying the associations of the change of TyG or BMI with CVD [19, 21]. Data‐driven approaches have better capacity to handle multidimensional and correlated data [19, 22, 23] and are therefore appropriate candidates for understanding the complex interrelationship between biomarkers and health outcomes. However, to date, the ways to capture the longitudinal co‐evolution of obesity, metabolic dysfunction, and inflammatory status remain underexplored, and no study has formally linked joint trajectories of BMI, TyG index, and hsCRP to subsequent cardiovascular endpoints.

Therefore, this study aims to address this critical knowledge and methodological gap. Leveraging a large‐scale, prospective longitudinal cohort, we integrate repeated measurements of the TyG index, BMI, and CRP, and apply a KmL3D trajectory clustering algorithm to identify distinct subtypes of “joint TyG‐BMI‐CRP evolution trajectories” within the population. Subsequently, we describe the characteristics of these trajectories. Finally, within a prospective follow‐up framework, we assess the association between different trajectory subtypes and subsequent incident CVD risk.

## 2 Materials and Methods

### 2.1 Study Design and Population

The China Health and Retirement Longitudinal Study (CHARLS) is a prospective and nationally representative cohort conducted in China [24]. The study collects data in repeated waves, with five waves currently available: Wave 1 (2011), Wave 2 (2013), Wave 3 (2015), Wave 4 (2018), and Wave 5 (2020). Detailed study designs are summarised in Supplementary methods 1. Wave 1 (2011) was regarded as the baseline, and Wave 3 (2015) was used as the second survey to evaluate the joint trajectories of BMI, TyG index, and hsCRP. Subsequent follow‐up surveys were used to track outcomes until the final follow‐up survey at Wave 5 (2020). The CHARLS was approved by the Ethics Review Committee of Peking University (IRB00001052‐11015), and informed consent was obtained from all participants.

**Figure 1:**
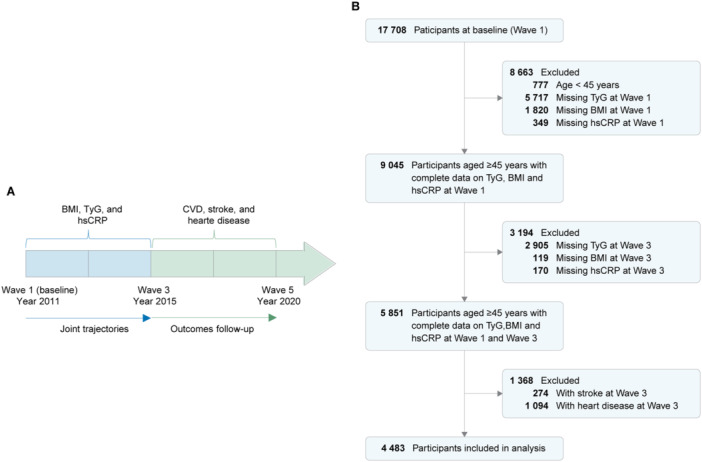
Study timeline and design (A) and selection process of population (B). BMI, body mass index; hsCRP, high‐sensitivity C‐reactive protein; TyG, triglyceride‐glucose index.

Figure 1B shows the flow of participant selection for the longitudinal analysis. Initially, 17 708 participants were enrolled at baseline (Wave 1). Among them, 8663 participants were excluded due to age < 45 years or missing data on TyG index, BMI, or hsCRP at baseline. At Wave 3, a further 3194 participants were excluded due to missing data on these same biomarkers. Then, 1368 participants with stroke or heart disease at Wave 3 were excluded. Finally, a total of 4483 participants were included in the final analysis.

### 2.2 Assessment of Joint Trajectories of BMI, TyG Index, and hsCRP

In the CHARLS study, BMI was calculated based on objectively measured height and weight data collected by trained interviewers during household visits. Height was measured to the nearest 0.1 cm using a portable stadiometer, and weight was measured to the nearest 0.1 kg using a digital scale, with participants wearing light indoor clothing and no shoes. BMI was then calculated as weight in kilograms divided by height in meters squared (kg/m^2^) [25]. Venous blood samples were collected from all participants by trained healthcare professionals during standardised in‐home visits. Following an overnight fast of at least 8 h, blood was drawn, processed, aliquoted, and stored at −80°C before being transported to a certified central laboratory for analysis. For the TyG index, serum triglyceride and fasting glucose concentrations were measured, and the TyG index was calculated as ln (fasting triglycerides [mg/dL] × fasting glucose [mg/dL]/2). Serum hsCRP was measured using a high‐sensitivity immunoturbidimetric assay.

**Figure 2:**
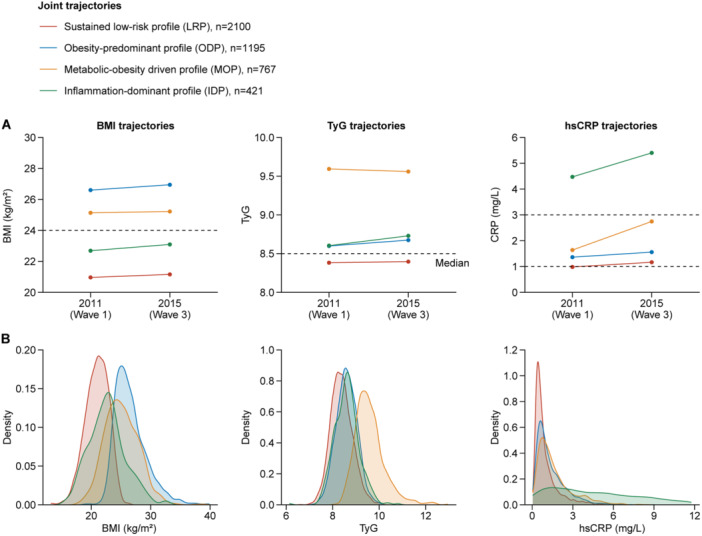
The best joint trajectories of BMI, TyG, and hsCRP. (A) Joint trajectories of BMI, TyG index, and hsCRP between Wave 1 and Wave 3. (B) Distribution of BMI, TyG, and hsCRP across joint trajectory groups. BMI, body mass index; hsCRP, high‐sensitivity C‐reactive protein; TyG, triglyceride‐glucose index.

Using the Kml3D algorithm [22, 23], a partitioning clustering method, we fitted models for 2 to 5 joint trajectory groups (Supporting Information S1: Figure 1). Finally, we identified four distinct joint trajectory groups as optimal by the max Calinski & Harabasz criterion (Supporting Information S1: Table 1). The four distinct joint trajectories of BMI, TyG index, and hsCRP from Wave 1 (2011) to Wave 3 (2015) are illustrated in Figure 2. The four identified joint trajectory groups are defined as follows [1]: sustained low‐risk profile (LRP), the largest group, with consistently low and stable levels of all biomarkers [2]; obesity‐predominant profile (ODP), featured by prominent and increasing BMI, with relatively stable and low TyG and CRP levels [3]; metabolic‐obesity driven profile (MOP), defined by the highest TyG level, overweight, and moderate CRP; and [4] inflammation‐dominant profile (IDP), characterised by the highest and rising CRP levels, with moderate BMI and TyG levels.

### 2.3 Outcome Ascertainment

The primary outcome for this study was incident CVD. Consistent with previous studies [26, 27], CVD was ascertained based on the self‐reported physician‐diagnosed heart disease or stroke. In each wave, participants were asked “Have you been told by a doctor that you have been diagnosed with a heart disease, including angina, heart attack, congestive heart failure, and other heart problems?” and “Have you been told by a doctor that you have been diagnosed with a stroke?” Those who reported being diagnosed with heart disease or stroke were considered to have CVD. In the next wave, participants were required to confirm the existence of heart disease and stroke if they reported those in the last wave. If participants disputed self‐reported heart disease or stroke from previous waves, they were considered not to have the disease. Follow‐up time was calculated from the date of the Wave 3 examination to the date of the first CVD event (stroke or heart disease), death, loss to follow‐up, or the end of the study period, whichever occurred first.

### 2.4 Covariates

The following covariates were included in the analyses: demographic characteristics, namely age (years, continuous), gender (male or female), marital status (married or other), and education level (classified as below high school, high school, college or above); socioeconomic and lifestyle factors, including smoking status (never, former, or current), drinking status (never, former, or current), nighttime sleep duration (hours, continuous), and depressive symptom score (continuous); as well as clinical and biomarker measures, such as hypertension, diabetes, dyslipidemia, kidney disease, use of antihypertensive medication, use of diabetes medication, use of lipid‐lowering medication, systolic and diastolic blood pressure (SBP/DBP; mmHg, continuous), high‐density lipoprotein cholesterol (HDL‐C; mg/dL, continuous), low‐density lipoprotein cholesterol (LDL‐C; mg/dL, continuous), estimated glomerular filtration rate (eGFR; mL/min/1.73 m^2^, continuous), glycated hemoglobin (HbA1c; %, continuous), total cholesterol (mg/dL, continuous), and triglycerides (mg/dL, continuous).

### 2.5 Statistical Analyses

To identify potential joint trajectories of BMI, TyG index, and hsCRP, this study employed KmL3D [22, 23], a partitioning clustering algorithm that extends the k‐means method [28]. The optimal number of clusters was determined by considering both the Calinski‐Harabasz criterion (where higher values indicate better partition) [22] and the clinical interpretability of the resulting trajectories. The derived clusters were subsequently labeled for use in subsequent analyses. Further methodological details are provided in Supporting Information Methods 3.

Continuous variables are presented as mean (standard deviation) for normally distributed data or median (interquartile range) for skewed data; categorical variables are presented as frequencies (percentages). Group comparisons for baseline variables were conducted using one‐way analysis of variance (ANOVA) for normally distributed continuous variables, the Kruskal−Wallis test for non‐normally distributed continuous variables, and the chi‐square test (or Fisher's exact test where appropriate) for categorical variables. Multinomial logistic regression was employed to identify baseline factors independently associated with membership in the three adverse trajectory subgroups. The LRP group served as the reference category. Unadjusted and adjusted odds ratios (ORs) with corresponding 95% confidence intervals (CIs) were reported.

Associations of baseline biomarkers (BMI, TyG index, and hsCRP) and joint trajectory group membership with incident CVD outcomes were evaluated using Cox proportional hazards regression models. Follow‐up time was calculated from the date of the baseline examination to the date of the first CVD event (stroke or heart disease), death, loss to follow‐up, or the end of the study period, whichever occurred first. The proportional hazards assumption was confirmed by visual inspection of log‐log plots and by testing Schoenfeld residuals, with no significant violations detected. For the analysis of individual biomarkers, baseline BMI, TyG index, and hsCRP were analyzed as categorical variables based on quartiles. For the primary analysis, joint trajectory group membership was entered as a categorical variable with the LRP as the reference group. Three nested models were constructed: Model 1 (unadjusted); Model 2 (adjusted for demographic and lifestyle factors); and Model 3 (additionally adjusted for clinical factors and medication use). For stroke and heart disease, the same modeling strategy was applied using the corresponding events as the outcome.

Subgroup analyses were conducted to assess the consistency of the association between joint trajectory groups and the primary CVD outcome across strata of key baseline characteristics. Heterogeneity of associations across subgroups was tested by introducing an interaction term between the trajectory group variable and the stratification variable into the fully adjusted Cox model. A series of sensitivity analyses were performed to evaluate the robustness of the primary findings [1]: a complete‐case analysis excluding participants with any missing covariate data [2]; further adjustment of Model 3 for additional potential confounders (SBP, DBP, HDL‐C, LDL‐C, eGFR, and HbA1c) [3]; exclusion of participants who developed a CVD event within the first 2 years of follow‐up to account for potential reverse causation; and [4] an analysis using multiple imputed datasets to assess the impact of missing data.

Missing data in baseline covariates were handled using multiple imputation by chained equations (MICE) [29]. Five imputed data sets were created, and estimates were pooled using Rubin's rules [29]. All statistical tests were two‐sided, and a *p* value < 0.05 was considered statistically significant. All analyses were conducted with R (version 4.5.1); specifically, we used “kml3d,” “nnet,” and “ggplot2” packages.

## 3 Results

### 3.1 Baseline Characteristics

Baseline characteristics are presented in Table 1; the study cohort (*N* = 4483) was stratified into four distinct profiles: the LRP (*n* = 2100, 46.8%), the ODP (*n* = 1195, 26.7%), the MOP (*n* = 767, 17.1%), and the IDP (*n* = 421, 9.4%). Significant differences (*p* < 0.05) were observed across these profiles for the majority of demographic, lifestyle, and clinical characteristics. Demographically, the IDP group was the oldest (mean age: 60.17 years), while the ODP group was the youngest (56.08 years). The proportion of women was highest in the ODP group (62.1%) and lowest in the IDP group (43.0%). A higher percentage of participants in the ODP and MOP groups were married. Educational attainment was generally low, with over 88% of participants in all groups having an education level below high school. Rural residence predominated overall but was less common in the ODP and MOP groups. Lifestyle patterns also differed: the ODP group reported the longest sleep duration and lowest depressive symptom scores, whereas current smoking was most prevalent in the IDP group.

**Table 1:** Baseline characteristics according to joint trajectories of BMI, triglyceride‐glucose index and high‐sensitivity C‐reactive protein.

Characteristics	LRP (*n* = 2100)	ODP (*n* = 1195)	MOP (*n* = 767)	IDP (*n* = 421)	*p* value
Age (years)	59.17 ± 8.80	56.08 ± 7.91	56.91 ± 7.93	60.17 ± 8.84	< 0.001^a^
Women	1004 (47.8%)	742 (62.1%)	449 (58.5%)	181 (43.0%)	< 0.001^b^
Married	1780 (84.8%)	1064 (89.0%)	672 (87.6%)	359 (85.3%)	0.004^b^
Education level					< 0.001^b^
Below high school	1942 (92.5%)	1053 (88.1%)	693 (90.4%)	396 (94.1%)	
High school	144 (6.9%)	128 (10.7%)	65 (8.5%)	24 (5.7%)	
College and above	14 (0.7%)	14 (1.2%)	9 (1.2%)	1 (0.2%)	
Rural residence	1549 (73.8%)	745 (62.3%)	450 (58.7%)	283 (67.2%)	< 0.001^b^
Drinking status					0.001^b^
Never	1169 (55.7%)	751 (62.8%)	466 (60.8%)	233 (55.3%)	
Former	153 (7.3%)	84 (7.0%)	46 (6.0%)	38 (9.0%)	
Current	778 (37.0%)	360 (30.1%)	255 (33.2%)	150 (35.6%)	
Smoking status					< 0.001^b^
Never	1177 (56.0%)	850 (71.1%)	502 (65.4%)	222 (52.7%)	
Former	142 (6.8%)	96 (8.0%)	52 (6.8%)	36 (8.6%)	
Current	781 (37.2%)	249 (20.8%)	213 (27.8%)	163 (38.7%)	
Night sleep duration (h)	6.32 ± 1.92	6.50 ± 1.80	6.46 ± 1.80	6.38 ± 1.83	0.050^a^
Depressive symptoms score	8.56 ± 6.29	7.46 ± 6.04	7.45 ± 5.94	8.54 ± 6.39	< 0.001^a^
Hypertension	251 (12.0%)	337 (28.2%)	236 (30.8%)	96 (22.8%)	< 0.001^b^
Diabetes	59 (2.8%)	50 (4.2%)	103 (13.4%)	17 (4.0%)	< 0.001^b^
Dyslipidemia	75 (3.6%)	113 (9.5%)	113 (14.7%)	27 (6.4%)	< 0.001^b^
Kidney disease	96 (4.6%)	54 (4.5%)	30 (3.9%)	21 (5.0%)	0.831^b^
Taking any hypertension medication	151 (7.2%)	243 (20.3%)	188 (24.5%)	62 (14.7%)	< 0.001^b^
Taking any diabetes medication	31 (1.5%)	28 (2.3%)	70 (9.1%)	10 (2.4%)	< 0.001^b^
Taking any medication for dyslipidemia	31 (1.5%)	54 (4.5%)	57 (7.4%)	15 (3.6%)	< 0.001^b^
SBP (mmHg)	124.02 ± 19.41	130.13 ± 20.92	131.92 ± 19.66	131.10 ± 22.50	< 0.001^a^
DBP (mmHg)	72.04 ± 11.42	77.31 ± 12.05	77.67 ± 11.55	75.66 ± 12.18	< 0.001^a^
HDL‐C (mg/dL)	57.22 ± 15.30	49.82 ± 12.63	39.89 ± 11.69	50.12 ± 14.18	< 0.001^a^
LDL‐C (mg/dL)	114.16 ± 31.72	120.27 ± 32.64	113.51 ± 41.81	121.05 ± 37.06	< 0.001^a^
eGFR (mL/min/1.73m^2^)	93.57 ± 13.33	95.27 ± 12.96	93.09 ± 14.65	90.42 ± 14.45	< 0.001^a^
HbA1c (%)	5.13 ± 0.60	5.18 ± 0.48	5.70 ± 1.34	5.21 ± 0.70	< 0.001^a^
BMI (kg/m^2^)	20.96 ± 1.95	26.60 ± 2.69	25.13 ± 2.84	22.69 ± 3.06	< 0.001^a^
TyG	8.38 ± 0.47	8.60 ± 0.45	9.59 ± 0.64	8.60 ± 0.53	< 0.001^a^
hsCRP (mg/L)	0.66 (0.41, 1.17)	1.02 (0.59, 1.81)	1.22 (0.70, 2.09)	4.04 (1.71, 6.73)	< 0.001^c^

*Note:* Data are mean ± SD, median (IQR) or *n* (%), unless otherwise specified.

Abbreviations: BMI, body mass index; DBP, diastolic blood pressure; eGFR, estimated glomerular filtration rate; HbA1c, hemoglobin A1c; HDL‐C, high‐density lipoprotein cholesterol; hsCRP, high‐sensitivity C‐reactive protein; IDP, inflammation‐dominant profile; IQR, interquartile range; LDL‐C, low‐density lipoprotein cholesterol; LRP, sustained low‐risk profile; MOP, metabolic‐obesity driven profile; ODP, obesity‐predominant profile; SBP, systolic blood pressure; SD, standard deviation; TyG, triglyceride‐glucose index.

^a^ Calculated by one‐way analysis of variance.

^b^ Calculated by Pearson's Chi‐squared test.

^c^ Calculated by Kruskal−Wallis rank sum test.

As expected from the profile definitions, distinct patterns were observed for the defining biomarkers. The LRP group had the lowest mean BMI (20.96 kg/m^2^), while the ODP group had the highest (26.60 kg/m^2^). The TyG index was highest in the MOP group (9.59). Median hsCRP levels were substantially elevated in the IDP group (4.04 mg/L) compared to the others. In addition, we described the baseline characteristics of participants using data not being imputed (Supporting Information S1: Table 2); these results were similar with Table 1.

### 3.2 Factors Associated With Joint Trajectory Group Membership

Multinomial logistic regression analysis revealed distinct factors associated with membership in the three adverse joint trajectory profiles compared to the LRP (Supporting Information S1: Table 3). For the ODP, female gender (OR = 1.46, 95% CI: 1.15–1.84) and urban residence were significant demographic predictors, whereas older age (OR = 0.96, 95% CI: 0.95–0.97) and current smoking (OR = 0.58, 95% CI: 0.45–0.73) were associated with lower odds. Clinically, hypertension showed the strongest association (OR = 3.26, 95% CI: 2.68–3.96), followed by dyslipidemia (OR = 2.02, 95% CI: 1.47–2.78). Similarly, for the MOP, female gender (OR = 1.52, 95% CI: 1.15–2.00) and urban residence remained key factors, while older age was also protective. However, this profile was most strongly characterised by clinical metabolic conditions, exhibiting the highest prevalence of diabetes, dyslipidemia, and related medication use. For the IDP, in contrast, the dominant predictors were current smoking (OR = 1.62, 95% CI: 1.23–2.13) and depressive symptoms (OR = 1.03, 95% CI: 1.01–1.05), with hypertension again significant but less dominant than in the ODP or MOP.

### 3.3 Associations of Baseline BMI, TyG Index, and hsCRP With Incident CVD

Supporting Information S1: Table 4 presents the association of baseline BMI, TyG index, and hsCRP with the incidence of CVD, stroke, and heart disease. After full adjustment for demographic, lifestyle, and clinical factors (Model 3), the HRs for the highest versus the lowest quartile of each marker were 1.44 (95% CI: 1.17–1.78) for BMI, 1.26 (95% CI: 1.03–1.54) for the TyG index, and 1.28 (95% CI: 1.05–1.55) for hsCRP in relation to total CVD. For stroke, the corresponding HRs were 1.59 (95% CI: 1.13–2.26) for BMI, 1.74 (95% CI: 1.23–2.46) for the TyG index, and 1.57 (95% CI: 1.15–2.15) for hsCRP. Regarding heart disease, only the highest BMI quartile showed a significant association (HR = 1.38, 95% CI: 1.07–1.76), while the associations for the highest quartiles of TyG index and hsCRP were not statistically significant (*p* > 0.05).

### 3.4 Association of Joint Trajectories of BMI, TyG Index, and hsCRP With Incident CVD

Table 2 presents the joint trajectories of BMI, TyG index, and hsCRP were significantly associated with the incidence of CVD, stroke, and heart disease during the follow‐up period. For the primary outcome of incidence of CVD, all three risk profiles showed significantly elevated risk compared to the LRP group in the fully adjusted model (Model 3). The ODP group was associated with a 35.0% increased risk (HR = 1.35, 95% CI: 1.13–1.60), the MOP group with a 41.0% increased risk (HR = 1.41, 95% CI: 1.15–1.71), and the IDP group with a 46.0% increased risk (HR = 1.46, 95% CI: 1.16–1.84). For stroke‐specific outcomes, the associations were even more pronounced. The MOP group demonstrated the highest risk (HR = 1.83, 95% CI: 1.34–2.50), followed by the IDP group (HR = 1.75, 95% CI: 1.22–2.50) and the ODP group (HR = 1.56, 95% CI: 1.14–2.14). For heart disease, the ODP, MOP, and IDP groups were associated with HRs of 1.29 (95% CI: 1.06–1.58), 1.25 (95% CI: 1.00–1.56), and 1.38 (95% CI: 1.07–1.79), respectively.

**Table 2:** Association between joint trajectories of body mass index, triglyceride‐glucose index and high‐sensitivity C‐reactive protein and incidence of cardiovascular diseases.

Outcomes	No. of event/ total	Model 1^a^		Model 2^b^		Model 3^c^	
		HR (95% CI)	*p* value	HR (95% CI)	*p* value	HR (95% CI)	*p* value
CVD							
LRP	316/2100	1 (Reference)		1 (Reference)		1 (Reference)	
ODP	252/1195	1.46 (1.23–1.73)	< 0.001	1.46 (1.23–1.74)	< 0.001	1.35 (1.13–1.60)	0.001
MOP	176/767	1.65 (1.37–1.99)	< 0.001	1.66 (1.38–2.00)	< 0.001	1.41 (1.15–1.71)	0.001
IDP	95/421	1.56 (1.24–1.97)	< 0.001	1.56 (1.24–1.97)	< 0.001	1.46 (1.16–1.84)	0.001
Stroke							
LRP	110/2100	1 (Reference)		1 (Reference)		1 (Reference)	
ODP	94/1195	1.70 (1.28–2.25)	< 0.001	1.69 (1.27–2.25)	< 0.001	1.50 (1.12–2.01)	0.006
MOP	79/767	2.28 (1.70–3.07)	< 0.001	2.29 (1.71–3.08)	1.83 (1.34–2.50)	< 0.001
IDP	42/421	1.92 (1.34–2.74)	< 0.001	1.92 (1.34–2.74)	< 0.001	1.75 (1.22–2.50)	0.002
Heart disease							
LRP	230/2100	1 (Reference)		1 (Reference)		1 (Reference)	
ODP	183/1195	1.38 (1.13–1.68)	0.001	1.38 (1.13–1.69)	0.001	1.29 (1.05–1.59)	0.015
MOP	116/767	1.42 (1.14–1.79)	0.002	1.44 (1.14–1.80)	0.002	1.25 (0.98–1.58)	0.070
IDP	62/421	1.41 (1.06–1.86)	0.017	1.41 (1.07–1.87)	0.016	1.33 (1.00–1.76)	0.048

Abbreviations: CI, confidence interval; CVD, cardiovascular diseases; HR, hazard ratio; IDP, inflammation‐dominant profile; LRP, sustained low‐risk profile; MOP, metabolic‐obesity driven profile; ODP, obesity‐predominant profile.

^a^ Adjusted for age, gender, marital status, education level, and residence type.

^b^ Adjusted for age, gender, marital status, education level, residence type, drinking status, smoking status, and night sleep duration.

^c^ Adjusted for age, gender, marital status, education level, residence type, drinking status, smoking status, night sleep duration, depressive symptoms score, hypertension diagnosis, diabetes diagnosis, dyslipidemia diagnosis, kidney disease diagnosis, taking any hypertension medication, taking any diabetes medication, and taking any medication for dyslipidemia.

### 3.5 Subgroup and Sensitivity Analyses

Subgroup analyses (Table 3, Supporting Information S1: 5 and 6) revealed that the associations between the joint trajectories of BMI, TyG index, and hsCRP and the incidence of CVD, stroke, and heart disease were generally consistent across various subgroups stratified by demographic, lifestyle, and clinical factors (All P_interaction_ > 0.05). Sensitivity analyses also remained consistent results. The associations between the joint trajectories of BMI, TyG index, and hsCRP and the incidence of CVD were materially unchanged in the subpopulation of 4133 participants with complete data (Supporting Information S1: Table 7), after further adjustment for additional covariates (Supporting Information S1: Table 8), and after excluding participants who developed CVD within the first 2 years of follow‐up (Supporting Information S1: Table 9). Notably, the association pattern also persisted in an analysis using multiply imputed data.

**Table 3:** Association between joint trajectories of body mass index, triglyceride‐glucose index and high‐sensitivity C‐reactive protein and incidence of cardiovascular diseases stratified by different factors.

Subgroup	No. of event/ total	Joint trajectories, HR (95% CI)^a^				P for interaction^a^
		LRP	ODP	MOP	IDP	
Age						0.239
45−59 years	429/2680	1 (Reference)	1.38 (1.09–1.76)	1.29 (0.98–1.71)	1.76 (1.26–2.46)	
≥60 years	410/1803	1 (Reference)	1.31 (1.01–1.70)	1.52 (1.14–2.02)	1.26 (0.91–1.73)	
Gender						0.924
Men	347/2107	1 (Reference)	1.48 (1.12–1.96)	1.56 (1.13–2.15)	1.45 (1.04–2.02)	
Women	492/2376	1 (Reference)	1.25 (1.00–1.56)	1.30 (1.01–1.68)	1.46 (1.06–2.02)	
*Marital status*						0.156
Married	726/3875	1 (Reference)	1.44 (1.19–1.74)	1.47 (1.19–1.83)	1.56 (1.21–2.00)	
Other	113/608	1 (Reference)	0.78 (0.45–1.35)	1.12 (0.64–1.96)	0.94 (0.50–1.77)	
*Education level*						0.261
Below high school	766/4084	1 (Reference)	1.33 (1.11–1.60)	1.39 (1.13–1.70)	1.49 (1.18–1.89)	
High school	68/361	1 (Reference)	1.82 (0.98–3.39)	1.73 (0.81–3.69)	1.64 (0.59–4.54)	
*Residence type*						0.772
Urban	271/1456	1 (Reference)	1.44 (1.05–1.99)	1.55 (1.11–2.18)	1.50 (0.97–2.32)	
Rural	568/3027	1 (Reference)	1.32 (1.07–1.63)	1.33 (1.04–1.71)	1.47 (1.12–1.93)	
*Drinking status*						0.822
Never	505/2619	1 (Reference)	1.27 (1.01–1.59)	1.24 (0.96–1.60)	1.39 (1.02–1.89)	
Former	76/321	1 (Reference)	1.60 (0.85–3.00)	1.31 (0.62–2.79)	1.65 (0.80–3.40)	
Current	258/1543	1 (Reference)	1.42 (1.03–1.96)	1.83 (1.29–2.59)	1.54 (1.02–2.32)	
*Smoking status*						0.150
Never	545/2751	1 (Reference)	1.29 (1.04–1.60)	1.33 (1.04–1.70)	1.47 (1.08–1.99)	
Former	68/326	1 (Reference)	1.44 (0.74–2.80)	0.98 (0.40–2.43)	2.52 (1.23–5.16)	
Current	226/1406	1 (Reference)	1.47 (1.03–2.09)	1.66 (1.14–2.41)	1.23 (0.80–1.88)	
*Hypertension*						0.900
No	586/3563	1 (Reference)	1.40 (1.14–1.72)	1.43 (1.12–1.81)	1.52 (1.16–2.00)	
Yes	253/920	1 (Reference)	1.16 (0.82–1.64)	1.25 (0.86–1.82)	1.23 (0.78–1.94)	
*Diabetes*						0.253
No	770/4254	1 (Reference)	1.39 (1.16–1.66)	1.34 (1.09–1.66)	1.46 (1.15–1.85)	
Yes	69/229	1 (Reference)	0.87 (0.36–2.10)	1.49 (0.74–2.99)	1.32 (0.44–3.89)	
*Dyslipidemia*						0.709
No	727/4155	1 (Reference)	1.38 (1.15–1.67)	1.47 (1.18–1.82)	1.48 (1.16–1.89)	
Yes	112/328	1 (Reference)	1.06 (0.60–1.88)	1.07 (0.62–1.84)	1.32 (0.63–2.74)	
*Kidney disease*						0.798
No	800/4282	1 (Reference)	1.34 (1.12–1.60)	1.38 (1.13–1.69)	1.45 (1.15–1.84)	
Yes	39/201	1 (Reference)	1.38 (0.57–3.34)	2.19 (0.79–6.08)	2.30 (0.72–7.30)	

Abbreviation: CI, confidence interval; HR, hazard ratio; IDP, inflammation‐dominant profile; LRP, sustained low‐risk profile; MOP, metabolic‐obesity driven profile; ODP, obesity‐predominant profile.

^a^ Adjusted for age, gender, marital status, education level, residence type, drinking status, smoking status, night sleep duration, depressive symptoms score, hypertension diagnosis, diabetes diagnosis, dyslipidemia diagnosis, kidney disease diagnosis, taking any hypertension medication, taking any diabetes medication, and taking any medication for dyslipidemia, but excluding stratified variable.

## 4 Discussion

This large‐scale, prospective cohort study employing data‐driven joint trajectory modeling revealed that the long‐term co‐evolutionary patterns of obesity, insulin resistance, and systemic inflammation define distinct cardiovascular risk phenotypes. We identified four mutually exclusive longitudinal profiles: a LRP, an ODP, a MOP, and an IDP. All three adverse profiles were independently associated with a significantly higher risk of incident CVD compared to the LRP. These findings underscore that the dynamic co‐evolution of obesity, insulin resistance, and systemic inflammation provides critical prognostic information beyond their static cross‐sectional measurements.

Our core finding that elevated levels of BMI, TyG index, and hsCRP are associated with increased cardiovascular risk aligns robustly with extensive existing literature [12, 13, 14, 15, 18]. Epidemiological studies have consistently established baseline obesity, insulin resistance, and chronic low‐grade inflammation as independent risk factors for subsequent cardiovascular events [12, 13, 14, 15, 16, 17]. Our observation that the highest quartiles of these individual biomarkers were associated with a 26%−44% increased risk of total CVD further corroborates their established pathogenic roles. The primary novelty of our study lies in the application of a longitudinal, multivariable trajectory clustering methodology. This represents a fundamental methodological departure from most prior studies that have examined these risk factors in isolation or at a single time point. By leveraging repeated measurements over a 4‐year period and jointly modeling three interrelated biological domains, our analysis reveals that distinct longitudinal phenotypes carry different prognostic weights.

The distinct trajectory subtypes identified likely reflect different underlying pathophysiological pathways and driving mechanisms in the development of atherosclerosis. The ODP likely represents a state of “metabolically healthy obesity” at baseline, where adipose tissue expansion has not yet triggered significant metabolic derangement or systemic inflammation. However, the elevated CVD risk in this group suggests that sustained obesity itself, perhaps through mechanical stress, altered adipokine secretion, or subclinical vascular dysfunction, poses an independent risk or represents a pre‐stage to more adverse profiles [30, 31]. The MOP epitomises the classic “adiposopathy” pathway [32]. Here, hypertrophic adipocytes become dysfunctional, leading to increased lipolysis, elevated free fatty acids, ectopic lipid deposition, and subsequent insulin resistance, which directly accelerates atherosclerosis through oxidative stress and endothelial dysfunction. The IDP, in turn, represents a predominantly inflammatory phenotype. This group may include individuals with occult infections, autoimmune tendencies, or chronic psychological stress, where systemic inflammation is the principal driver of vascular injury [33, 34, 35]. Importantly, all three profiles conferred increased CVD risk, but the magnitude and pattern differed: the MOP showed the strongest association with stroke, whereas the IDP showed the most pronounced risk for overall CVD.

Our findings have several important implications. From a clinical perspective, we argue for a paradigm shift from static to dynamic, multi‐system risk assessment. A single measurement of BMI, glucose, or CRP provides an incomplete profile. Instead, clinicians should consider the trajectory of these markers. Identifying a patient in the ODP trajectory could trigger intensive lifestyle interventions focused on weight management to prevent progression to the MOP. Detecting an individual in the IDP trajectory warrants investigation into sources of chronic inflammation. For those in the MOP, aggressive management of insulin resistance and lipids, alongside anti‐inflammatory strategies, may be paramount. From a public health standpoint, these profiles can help tailor population‐level screening and prevention strategies, particularly in resource‐limited settings where repeated measurement of simple, low‐cost biomarkers may offer more actionable information than expensive single‐time‐point assays.

The major strengths of this study include its large, prospective, and nationally representative cohort design, and the application of a novel data‐driven joint trajectory analysis. Nevertheless, several limitations should be acknowledged. First, consistent with previous studies [26, 27], CVD outcomes were based on self‐reported physician diagnoses, which may be subject to recall bias and underdiagnosis, although this method is commonly used in large epidemiological cohorts like ELSA [36]; it found that self‐reported incident coronary heart disease had a good agreement with medical records (accuracy, 77.5%). Second, the biomarkers were measured at only two time points (2011 and 2015) to define trajectories spanning 4 years. More frequent measurements would allow for more precise modeling of intra‐individual variability and could reveal transition phases not captured here. Third, although we adjusted for a wide range of confounders, residual confounding from unmeasured factors such as dietary quality, physical activity patterns, or genetic susceptibility cannot be excluded. Fourth, the CHARLS cohort is Chinese, and the generalisability of our findings to other ethnicities requires further validation.

## 5 Conclusions

In conclusion, this study demonstrates that the longitudinal joint trajectories of BMI, TyG index, and hsCRP identify distinct cardiometabolic phenotypes with differential risks for future CVD, thereby highlighting the critical importance of monitoring the dynamic interplay of obesity, metabolism, and inflammation over time for refined risk prediction. Future research should investigate the genetic, epigenetic, microbiome, and environmental determinants that drive individuals onto different trajectories.

## Author Contributions

**Qinggao Wang:** data curation, formal analysis, writing – original draft, writing – review and editing. **Yilu Lei:** data curation, formal analysis, writing – original draft, writing – review and editing. **Qingfeng Zhou:** writing – review and editing. **Yan Long:** writing – review and editing. **Yinjuan Lai:** conceptualization, writing – review and editing. All authors contributed to manuscript revision, read and approved the submitted version.

## Ethics Statement

The China Health and Retirement Longitudinal Study was approved by the Ethics Review Committee of Peking University (IRB00001052‐11015). All procedures performed in this study involving human participants were in accordance with the ethical standards of the institutional and national research committee and with the 1964 Helsinki Declaration and its later amendments or comparable ethical standards. Informed consent was obtained from each subject.

## Conflicts of Interest

The authors declare no conflicts of interest.

## Declaration of Generative AI and AI‐Assisted Technologies in the Writing Process

Generative AI (ChatGPT 5.0) was used solely for language polishing, grammar correction, and readability improvement of the manuscript text. The authors confirm that no AI was involved in the research design, data analysis, interpretation of results, or drafting of scientific content. All final revisions, editorial decisions, and responsibility for the accuracy and integrity of the work rest solely with the authors.

## Supporting information


Supporting File

## Data Availability

Data supporting the results of this study are available from the official website of China Health and Retirement Longitudinal Study (http://charls.pku.edu.en).
