# Supplementary material for: Dynamic Co‐Evolution of Obesity‐Metabolic‐Inflammatory for Cardiovascular Disease Risk Stratification in Middle‐Aged and Older Adults: A Data‐Driven Joint Trajectory Analysis
Source: Clin Cardiol. 2026 Jul 29;49(8):e70429. doi: 10.1002/clc.70429 (PMC13416755; doi:10.1002/clc.70429)
Supplement: Supplementary file 1 — Supporting File [file CLC-49-e70429-s001.docx]

Supplemental methods 1: Study designs of the CHARLS

The China Health and Retirement Longitudinal Study (CHARLS) was a prospective cohort study conducted in China. In wave 1, a nationally representative sample of 17,708 participants was recruited from 28 provinces in 2011 via multistage probability sampling. The primary aim of this study was to recruit participants aged ≥ 45 years, but some participants aged 40 to 44 years also attended the baseline survey. All 17,708 participants underwent face-to-face interviews by the trained staff using the standardized questionnaire to collect data on sociodemographic information, lifestyles, and health-related information. Among 17,708 participants, 13,978 participants conducted anthropometric measurements to collect data on height, weight, waist circumference, hip circumference, blood pressure, grip strength, and so on. In addition, 11,847 participants provided blood samples for the laboratory test. Biochemical indicators, including total cholesterol, high-density lipoprotein cholesterol, low-density lipoprotein cholesterol, glycated hemoglobin, fasting blood glucose, and C-reactive protein were measured by the blood test. The follow-up surveys were conducted in 2013 (wave 2), 2015 (wave 3), 2018 (wave 4), and 2020 (wave 5) with questionnaire interviews and anthropometric measurements. Blood samples were collected in 2015 again, and biochemical indicators were measured by the blood test.

**Supplementary methods 2:** Covariates

Depressive symptoms were measured using the CES-D short form, which is a widely used self-report measure on depressive symptoms in population-based studies. The estimated glomerular filtration rate was calculated using the Chronic Kidney Disease Epidemiology Collaboration’s 2009 creatinine equation. Hypertension defined as systolic blood pressure ≥140 mm Hg, diastolic blood pressure ≥90 mm Hg, current use of the antihypertensive medication, or self-reported history of hypertension. Diabetes (defined as fasting plasma glucose ≥126 mg/dL [to convert to millimoles per liter, multiply by 0.0555], current use of antidiabetic medication, or self-reported history of diabetes). Dyslipidemia defined as total cholesterol ≥240 mg/dL [to convert to millimoles per liter, multiply by 0.0259], triglycerides ≥150 mg/dL, low-density lipoprotein cholesterol ≥160 mg/dL, high-density lipoprotein cholesterol <40 mg/dL, current use of lipid-lowering medication, or self-reported history of dyslipidemia. Chronic kidney disease defined as estimated glomerular filtration rate <60 mL/min/1.73 m^2^ or self-reported history of chronic kidney disease.

**Supplementary methods 3:** Introduction to KmL3D algorithm

Our cohort study employs a longitudinal design, wherein the variables of obesity and depression are repeatedly measured over time. Given that both variables exhibit temporal fluctuations, it is feasible to model their respective “variable-trajectories” through longitudinal trajectory analysis, thereby capturing interindividual variability in developmental patterns across the study period. Usually, a standard way to analyzing variable-trajectories involves employing clustering algorithm to identify the existence of homogeneous developmental patterns. However, the traditional methods for exploring potential trajectory are based on the clustering of single variable-trajectory, without considering the possible co-evolution of variable-trajectories. Meanwhile, only focusing on single-disease trajectory may oversimplify the research questions and be far away from the reality of the coexistence of multiple diseases in real-world populations.

To address such methodological limitations, the present study employed the KmL3D clustering algorithm developed by Genolini, which a partitioning algorithm that can work jointly on multiple variable trajectories. It is an updated version of the widely used K-means clustering algorithm and enables simultaneous modeling of interdependent variable trajectories (called joint trajectories). KmL3D not only does incorporate functionality for handling missing data, but also offers several clustering quality criteria to evaluate partition validity (Calinski & Harabasz criterion is mainly considered and high value denoting good partition). And its graphic interface helps the user to select the best partition. The working principle is as follows: assuming these trajectories are interconnected, Kml3D algorithm clusters the data into K computer-selected or user-defined disjoint clusters based on the combined distances among variable trajectories. This ensures participants within the same group show the highest pairwise similarity scores while participants in different cluster groups show the highest dissimilarity scores.

Therefore, when determining the optimal number and shape of joint trajectories, we can divide trajectories by taking into account the recommended choice of computers based on algorithm-suggested criteria (e.g., Calinski-Harabasz criterion score), the proportion of the number of people in each trajectory group, theoretical relevance to research objectives, and pragmatic interpretability of resulting trajectory profiles.

**
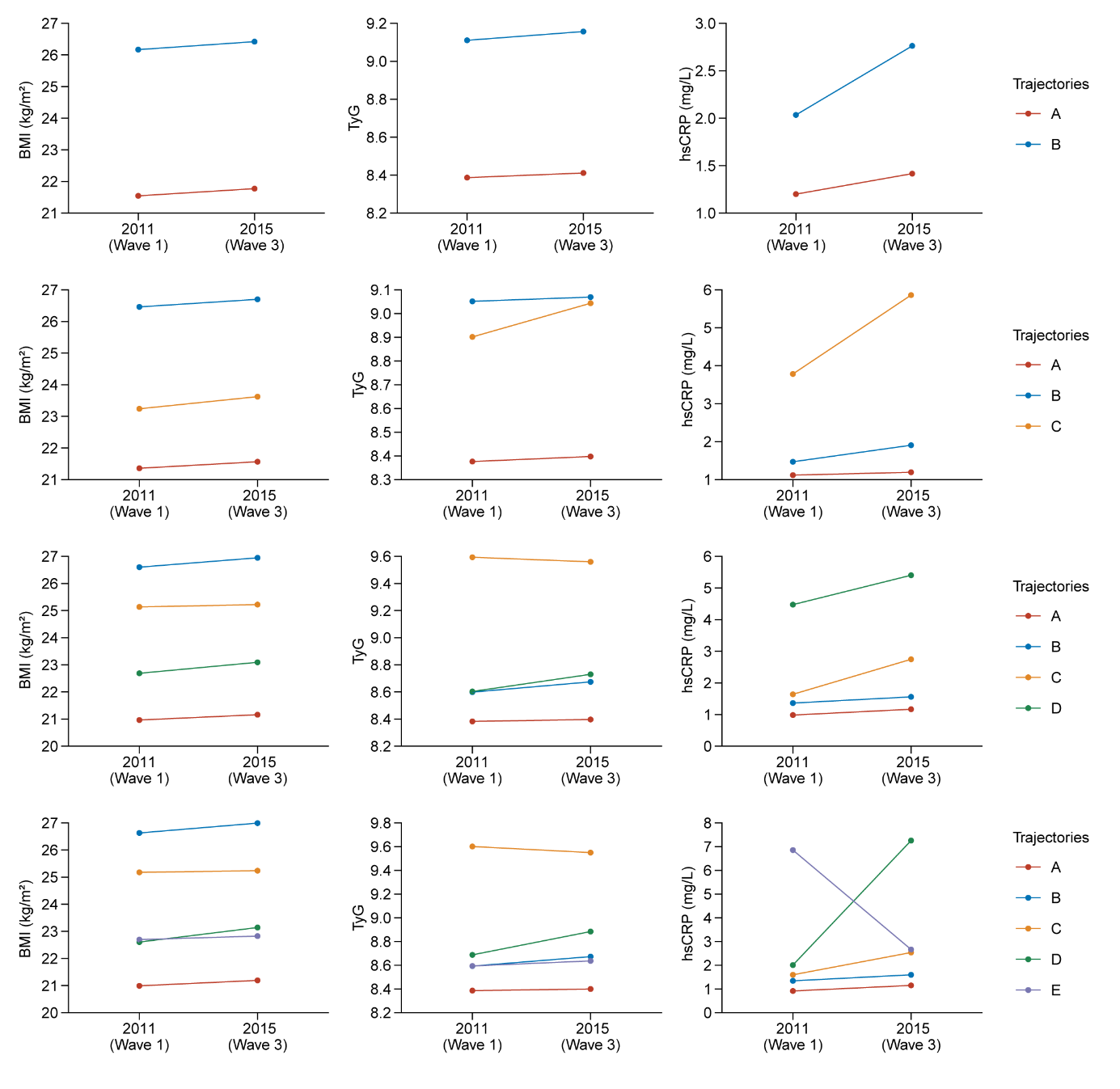
**

**Figure S1. Joint trajectories of body mass index, triglyceride-glucose index, and high-sensitivity C-reactive protein.**

Abbreviations: BMI, body mass index; hsCRP, high-sensitivity C-reactive protein; TyG, triglyceride-glucose index.

**
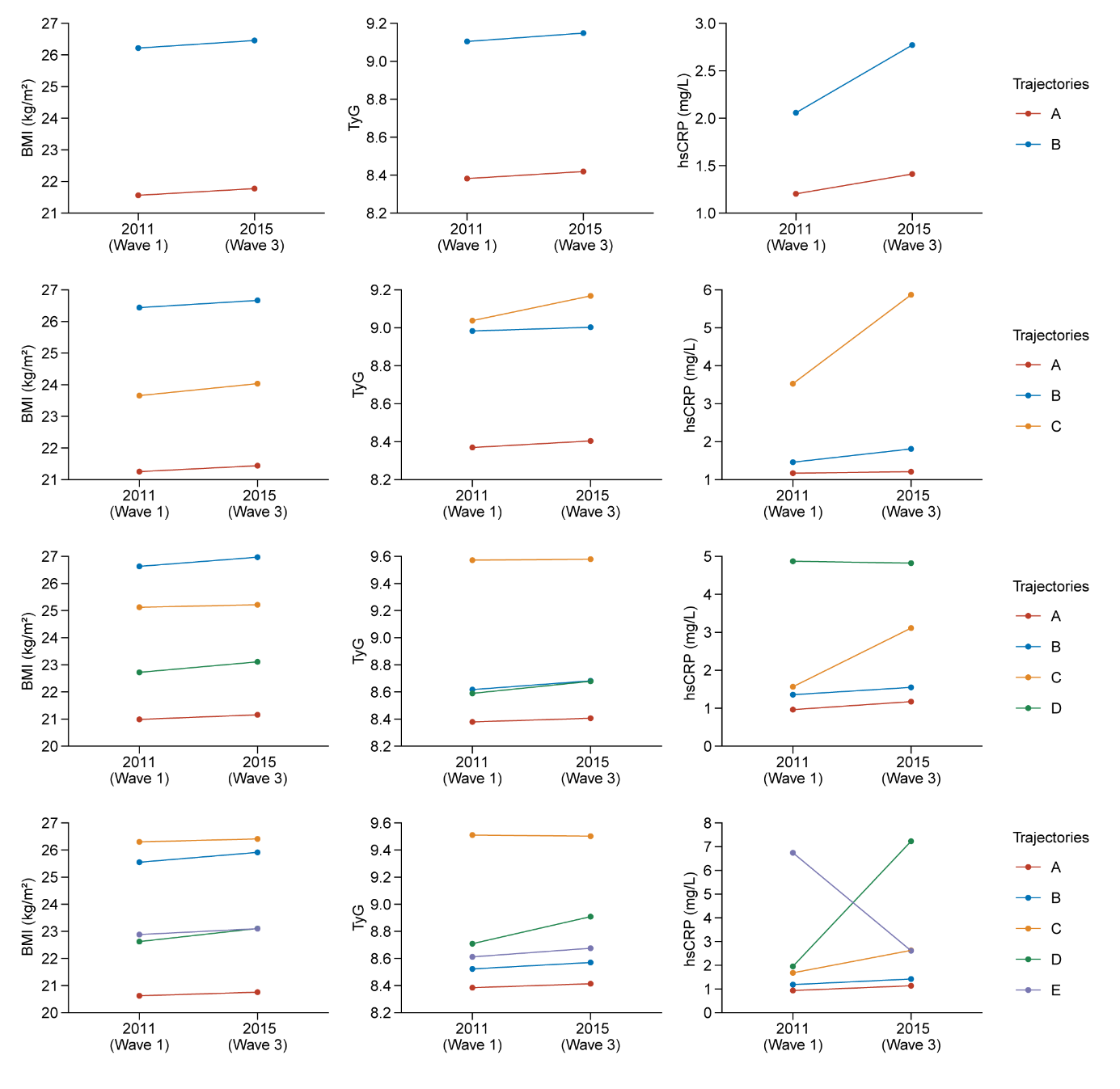
**

**Figure S2. Joint trajectories of body mass index, triglyceride-glucose index, and high-sensitivity C-reactive protein after random exclusion of 20% of participants.**

Abbreviations: BMI, body mass index; hsCRP, high-sensitivity C-reactive protein; TyG, triglyceride-glucose index.

**
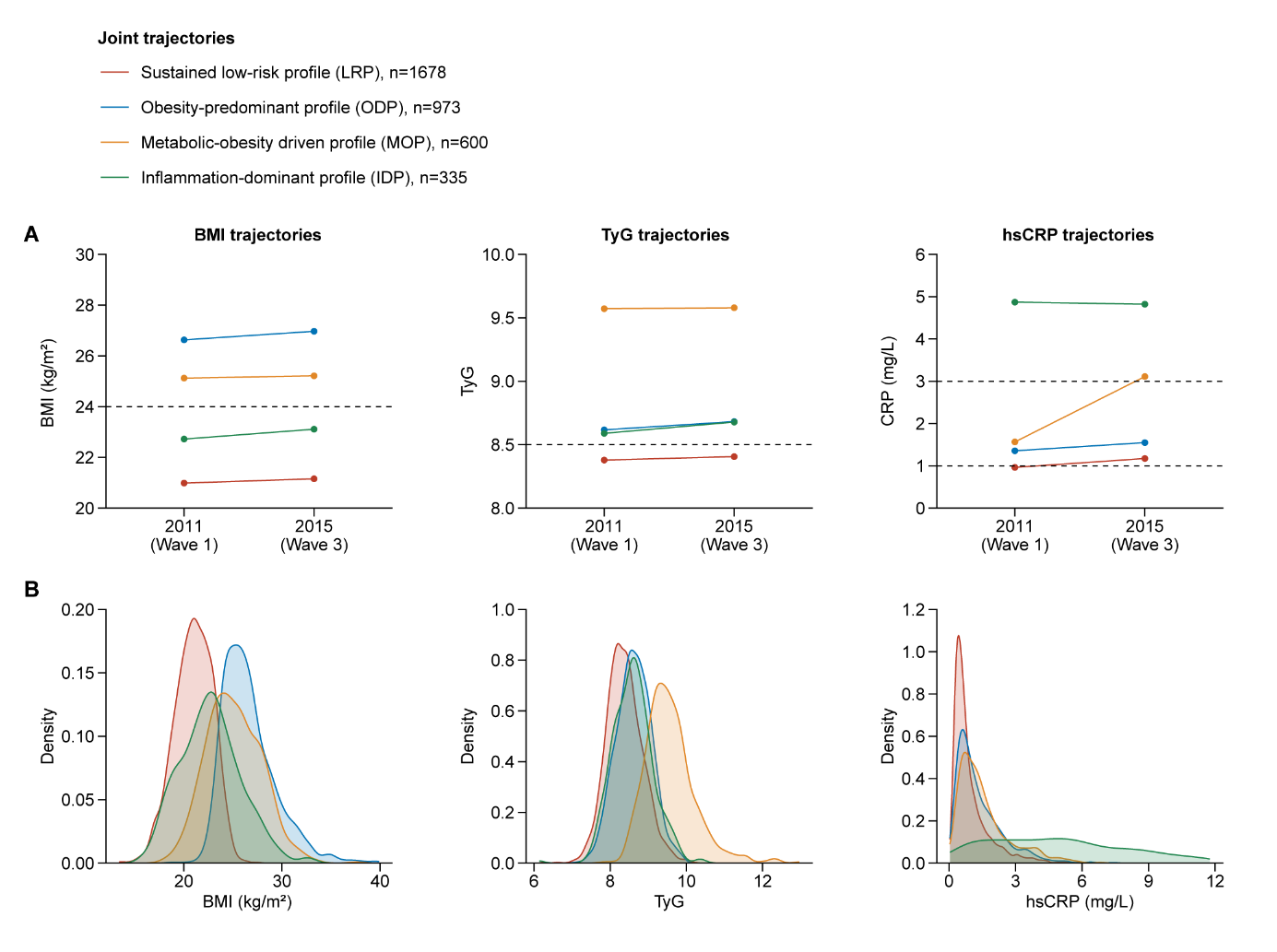
**

**Figure S3. The best joint trajectories of body mass index, triglyceride-glucose index, and high-sensitivity C-reactive protein after random exclusion of 20% of participants.**

(A) Joint trajectories of BMI, TyG index, and hs-CRP between Wave 1 and Wave 3. (B) Distribution of BMI, TyG, and hsCRP across joint trajectory groups.

Abbreviations: BMI, body mass index; hsCRP, high-sensitivity C-reactive protein; TyG, triglyceride-glucose index.

Table S1. Calinski & Harabasz criterion

| Number of trajectories | Calinski value ^a^ | Harabasz value ^a^ |
| --- | --- | --- |
| 2 | 1277.879 | 1277.747 |
| 3 | 1315.708 | 1314.336 |
| 4 | 1384.753 | 1384.674 |
| 5 | 1318.746 | 1318.949 |

^a^ High value denoting good partition.

Table S2. Baseline characteritics according to joint trajectories of body mass index, triglyceride-glucose index and high-sensitivity C-reactive protein (non-imputed data).

| Characteristics | LRP (n=2100) | ODP (n=1195) | MOP (n=767) | IDP (n=421) | P value |
| --- | --- | --- | --- | --- | --- |
| Age (years) | 59.17±8.80 | 56.08±7.91 | 56.91±7.93 | 60.17±8.84 | <0.001 ^a^ |
| Women | 1004 (47.8%) | 742 (62.1%) | 449 (58.5%) | 181 (43.0%) | <0.001 ^b^ |
| Married | 1780 (84.8%) | 1064 (89.0%) | 672 (87.6%) | 359 (85.3%) | 0.004 ^b^ |
| Education level |  |  |  |  | <0.001 ^b^ |
| Below high school | 1942 (92.5%) | 1053 (88.1%) | 693 (90.4%) | 396 (94.1%) |  |
| High school | 144 (6.9%) | 128 (10.7%) | 65 (8.5%) | 24 (5.7%) |  |
| College and above | 14 (0.7%) | 14 (1.2%) | 9 (1.2%) | 1 (0.2%) |  |
| Rural residence | 1549 (73.8%) | 745 (62.3%) | 450 (58.7%) | 283 (67.2%) | <0.001 ^b^ |
| Drinking status ^d^ |  |  |  |  | 0.001 ^b^ |
| Never | 1168 (55.6%) | 750 (62.8%) | 466 (60.8%) | 233 (55.3%) |  |
| Former | 153 (7.3%) | 84 (7.0%) | 46 (6.0%) | 38 (9.0%) |  |
| Current | 777 (37.0%) | 360 (30.1%) | 255 (33.2%) | 150 (35.6%) |  |
| Smoking status ^d^ |  |  |  |  | <0.001 ^b^ |
| Never | 1175 (56.0%) | 850 (71.1%) | 501 (65.3%) | 221 (52.5%) |  |
| Former | 141 (6.7%) | 96 (8.0%) | 51 (6.6%) | 36 (8.6%) |  |
| Current | 778 (37.0%) | 248 (20.8%) | 212 (27.6%) | 163 (38.7%) |  |
| Night sleep duration (h) ^d^ | 6.33±1.93 | 6.51±1.79 | 6.47±1.79 | 6.36±1.83 | 0.046 ^a^ |
| Depressive symptoms score ^d^ | 8.55±6.25 | 7.48±6.03 | 7.43±5.94 | 8.46±6.42 | <0.001 ^a^ |
| Hypertension ^d^ | 251 (12.0%) | 336 (28.1%) | 233 (30.4%) | 96 (22.8%) | <0.001 ^b^ |
| Diabetes ^d^ | 59 (2.8%) | 49 (4.1%) | 102 (13.3%) | 17 (4.0%) | <0.001 ^b^ |
| Dyslipidemia ^d^ | 75 (3.6%) | 111 (9.3%) | 111 (14.5%) | 27 (6.4%) | <0.001 ^b^ |
| Kidney disease ^d^ | 95 (4.5%) | 53 (4.4%) | 30 (3.9%) | 21 (5.0%) | 0.840 ^b^ |
| Taking any hypertension medication ^d^ | 151 (7.2%) | 242 (20.3%) | 185 (24.1%) | 62 (14.7%) | <0.001 ^b^ |
| Taking any diabetes medication ^d^ | 30 (1.4%) | 27 (2.3%) | 69 (9.0%) | 10 (2.4%) | <0.001 ^b^ |
| Taking any medication for dyslipidemia ^d^ | 31 (1.5%) | 52 (4.4%) | 56 (7.3%) | 15 (3.6%) | <0.001 ^b^ |
| SBP (mmHg) ^d^ | 123.92±19.31 | 130.18±20.93 | 131.94±19.62 | 130.99±22.47 | <0.001 ^a^ |
| DBP (mmHg) ^d^ | 72.00±11.41 | 77.34±12.07 | 77.67±11.55 | 75.63±12.17 | <0.001 ^a^ |
| HDL-C (mg/dL) | 57.22±15.30 | 49.82±12.63 | 39.89±11.69 | 50.12±14.18 | <0.001 ^a^ |
| LDL-C (mg/dL) ^d^ | 114.16±31.72 | 120.27±32.64 | 113.66±41.53 | 121.05±37.06 | <0.001 ^a^ |
| eGFR (mL/min/1.73m^2^) ^d^ | 93.57±13.33 | 95.27±12.96 | 93.09±14.67 | 90.42±14.45 | <0.001 ^a^ |
| HbA1c (%) ^d^ | 5.13±0.60 | 5.18±0.48 | 5.71±1.34 | 5.21±0.70 | <0.001 ^a^ |
| BMI (kg/m^2^) | 20.96±1.95 | 26.60±2.69 | 25.13±2.84 | 22.69±3.06 | <0.001 ^a^ |
| TyG | 8.38±0.47 | 8.60±0.45 | 9.59±0.64 | 8.60±0.53 | <0.001 ^a^ |
| hsCRP (mg/L) | 0.66 (0.41, 1.17) | 1.02 (0.59, 1.81) | 1.22 (0.70, 2.09) | 4.04 (1.71, 6.73) | <0.001 ^c^ |

Data were mean±SD, median (IQR) or n(%), unless otherwise specified.

Abbreviations: BMI, body mass index; DBP, diastolic blood pressure; eGFR, estimated glomerular filtration rate; HbA1c, hemoglobin A1c; HDL-C, high-density lipoprotein cholesterol; hsCRP, high-sensitivity C-reactive protein; IDP, inflammation-dominant profile; IQR, interquartile range; LDL-C, low-density lipoprotein cholesterol; LRP, sustained low-risk profile; MOP, metabolic-obesity driven profile; ODP, obesity-predominant profile; SBP, systolic blood pressure; SD, standard deviation; TyG, triglyceride-glucose index.

^a^ Calculated by one-way analysis of variance.

^b^ Calculated by Pearson's Chi-squared test.

^c^ Calculated by Kruskal-Wallis rank sum test.

^d^ Missing data: 3 for drinking status, 11 for smoking status, 53 for night sleep duration, 238 for depressive symptoms score, 19 for hypertension, 32 for diabetes, 88 for dyslipidemia, 15 for kidney disease, 19 for taking any hypertension medication, 33 for taking any diabetes medication, 88 for taking any medication for dyslipidemia, 27 for SBP, 27 for DBP, 8 for LDL-C, 2 for eGFR, 32 for HbA1c.

**Table S3. Multinomial logistic regression of factors associated with joint trajectories group membership (*vs.* LRP)**

| Variable | ODP *vs*. LRP | |  | MOP *vs*. LRP | |  | IDP *vs*. LRP | |
| --- | --- | --- | --- | --- | --- | --- | --- | --- |
|  | OR (95% CI) | P value |  | OR (95% CI) | P value |  | OR (95% CI) | P value |
| Age (years) | 0.96 (0.95–0.97) | <0.001 |  | 0.97 (0.95–0.98) | <0.001 |  | 1.01 (1.00–1.02) | 0.208 |
| Gender |  |  |  |  |  |  |  |  |
| Men | Reference |  |  | Reference |  |  | Reference |  |
| Women | 1.46 (1.15–1.84) | 0.002 |  | 1.52 (1.15–2.00) | 0.003 |  | 0.77 (0.56–1.06) | 0.106 |
| Marital status |  |  |  |  |  |  |  |  |
| Married | Reference |  |  | Reference |  |  | Reference |  |
| Other | 0.80 (0.63–1.01) | 0.062 |  | 0.93 (0.72–1.21) | 0.607 |  | 0.92 (0.68–1.25) | 0.599 |
| Education level |  |  |  |  |  |  |  |  |
| Below high school | Reference |  |  | Reference |  |  | Reference |  |
| High school | 1.25 (0.95–1.64) | 0.105 |  | 0.94 (0.68–1.31) | 0.720 |  | 0.78 (0.49–1.24) | 0.290 |
| College and above | 1.58 (0.72–3.49) | 0.255 |  | 1.42 (0.58–3.47) | 0.441 |  | 0.29 (0.04–2.23) | 0.235 |
| Residence type |  |  |  |  |  |  |  |  |
| Urban | Reference |  |  | Reference |  |  | Reference |  |
| Rural | 0.66 (0.56–0.77) | <0.001 |  | 0.56 (0.47–0.67) | <0.001 |  | 0.70 (0.56–0.88) | 0.003 |
| Drinking status |  |  |  |  |  |  |  |  |
| Never | Reference |  |  | Reference |  |  | Reference |  |
| Former | 1.14 (0.83–1.56) | 0.417 |  | 0.86 (0.59–1.26) | 0.445 |  | 0.97 (0.65–1.46) | 0.896 |
| Current | 1.07 (0.88–1.30) | 0.507 |  | 1.14 (0.91–1.43) | 0.239 |  | 0.86 (0.66–1.13) | 0.278 |
| Smoking status |  |  |  |  |  |  |  |  |
| Never | Reference |  |  | Reference |  |  | Reference |  |
| Former | 1.24 (0.89–1.73) | 0.194 |  | 1.14 (0.76–1.71) | 0.515 |  | 1.14 (0.73–1.80) | 0.560 |
| Current | 0.58 (0.45–0.73) | <0.001 |  | 0.89 (0.68–1.16) | 0.383 |  | 1.00 (0.74–1.36) | 0.999 |
| Night sleep duration | 1.03 (0.98–1.07) | 0.246 |  | 1.02 (0.97–1.07) | 0.546 |  | 1.02 (0.97–1.08) | 0.420 |
| Depressive symptoms score | 0.97 (0.96–0.98) | <0.001 |  | 0.97 (0.95–0.98) | <0.001 |  | 1.00 (0.99–1.02) | 0.760 |
| Hypertension |  |  |  |  |  |  |  |  |
| No | Reference |  |  | Reference |  |  | Reference |  |
| Yes | 3.26 (2.68–3.96) | <0.001 |  | 3.15 (2.54–3.92) | <0.001 |  | 2.05 (1.56–2.68) | <0.001 |
| Diabetes |  |  |  |  |  |  |  |  |
| No | Reference |  |  | Reference |  |  | Reference |  |
| Yes | 1.08 (0.72–1.62) | 0.711 |  | 3.69 (2.58–5.28) | <0.001 |  | 1.18 (0.67–2.07) | 0.562 |
| Dyslipidemia |  |  |  |  |  |  |  |  |
| No | Reference |  |  | Reference |  |  | Reference |  |
| Yes | 2.02 (1.47–2.78) | <0.001 |  | 2.90 (2.09–4.02) | <0.001 |  | 1.64 (1.03–2.60) | 0.037 |
| Kidney disease |  |  |  |  |  |  |  |  |
| No | Reference |  |  | Reference |  |  | Reference |  |
| Yes | 1.02 (0.71–1.47) | 0.907 |  | 0.85 (0.55–1.32) | 0.469 |  | 1.07 (0.65–1.74) | 0.800 |

Abbreviations: IDP, inflammation-dominant profile; LRP, sustained low-risk profile; MOP, metabolic-obesity driven profile; ODP, obesity-predominant profile; OR, odds ratio; CI, confidence interval.

Table S4. Association of baseline body mass index, triglyceride-glucose index and high-sensitivity C-reactive protein with incidence of cardiovascular diseases

| Outcomes | No. of event /  total | Model 1 ^a^ | |  | Model 2 ^b^ | |  | Model 3 ^c^ | |
| --- | --- | --- | --- | --- | --- | --- | --- | --- | --- |
|  |  | HR (95% CI) | P value |  | HR (95% CI) | P value |  | HR (95% CI) | P value |
| CVD |  |  |  |  |  |  |  |  |  |
| BMI |  |  |  |  |  |  |  |  |  |
| Quartile 1 | 181 / 1153 | 1 (Reference) |  |  | 1 (Reference) |  |  | 1 (Reference) |  |
| Quartile 2 | 183 / 1103 | 1.08 (0.88–1.33) | 0.460 |  | 1.09 (0.89–1.34) | 0.416 |  | 1.09 (0.88–1.34) | 0.441 |
| Quartile 3 | 209 / 1131 | 1.21 (0.99–1.48) | 0.063 |  | 1.22 (1.00–1.50) | 0.053 |  | 1.17 (0.95–1.43) | 0.144 |
| Quartile 4 | 266 / 1096 | 1.67 (1.37–2.04) | <0.001 |  | 1.68 (1.38–2.06) | <0.001 |  | 1.44 (1.17–1.78) | 0.001 |
| TyG |  |  |  |  |  |  |  |  |  |
| Quartile 1 | 170 / 1121 | 1 (Reference) |  |  | 1 (Reference) |  |  | 1 (Reference) |  |
| Quartile 2 | 200 / 1121 | 1.17 (0.95–1.44) | 0.130 |  | 1.17 (0.96–1.44) | 0.126 |  | 1.13 (0.92–1.39) | 0.235 |
| Quartile 3 | 224 / 1120 | 1.28 (1.05–1.56) | 0.016 |  | 1.27 (1.04–1.55) | 0.019 |  | 1.20 (0.98–1.46) | 0.083 |
| Quartile 4 | 245 / 1121 | 1.46 (1.20–1.78) | <0.001 |  | 1.46 (1.20–1.77) | <0.001 |  | 1.26 (1.03–1.54) | 0.027 |
| hsCRP |  |  |  |  |  |  |  |  |  |
| Quartile 1 | 185 / 1145 | 1 (Reference) |  |  | 1 (Reference) |  |  | 1 (Reference) |  |
| Quartile 2 | 177 / 1112 | 1.00 (0.81–1.23) | 0.993 |  | 1.00 (0.82–1.23) | 0.985 |  | 0.99 (0.80–1.22) | 0.916 |
| Quartile 3 | 233 / 1106 | 1.29 (1.06–1.57) | 0.010 |  | 1.30 (1.07–1.57) | 0.009 |  | 1.24 (1.02–1.51) | 0.029 |
| Quartile 4 | 244 / 1120 | 1.39 (1.15–1.69) | 0.001 |  | 1.39 (1.14–1.68) | 0.001 |  | 1.28 (1.05–1.55) | 0.014 |
| Stroke |  |  |  |  |  |  |  |  |  |
| BMI |  |  |  |  |  |  |  |  |  |
| Quartile 1 | 65 / 1153 | 1 (Reference) |  |  | 1 (Reference) |  |  | 1 (Reference) |  |
| Quartile 2 | 68 / 1103 | 1.19 (0.85–1.68) | 0.319 |  | 1.20 (0.85–1.69) | 0.295 |  | 1.19 (0.84–1.67) | 0.331 |
| Quartile 3 | 90 / 1131 | 1.60 (1.16–2.22) | 0.004 |  | 1.62 (1.17–2.25) | 0.004 |  | 1.52 (1.09–2.11) | 0.014 |
| Quartile 4 | 102 / 1096 | 2.04 (1.47–2.82) | <0.001 |  | 2.05 (1.48–2.85) | <0.001 |  | 1.59 (1.13–2.26) | 0.009 |
| TyG |  |  |  |  |  |  |  |  |  |
| Quartile 1 | 53 / 1121 | 1 (Reference) |  |  | 1 (Reference) |  |  | 1 (Reference) |  |
| Quartile 2 | 76 / 1121 | 1.48 (1.04–2.10) | 0.028 |  | 1.47 (1.04–2.09) | 0.031 |  | 1.40 (0.98–1.99) | 0.062 |
| Quartile 3 | 91 / 1120 | 1.76 (1.25–2.47) | 0.001 |  | 1.74 (1.24–2.44) | 0.001 |  | 1.60 (1.13–2.25) | 0.007 |
| Quartile 4 | 105 / 1121 | 2.14 (1.54–2.99) | <0.001 |  | 2.13 (1.52–2.97) | <0.001 |  | 1.74 (1.23–2.46) | 0.002 |
| hsCRP |  |  |  |  |  |  |  |  |  |
| Quartile 1 | 64 / 1145 | 1 (Reference) |  |  | 1 (Reference) |  |  | 1 (Reference) |  |
| Quartile 2 | 64 / 1112 | 1.05 (0.74–1.48) | 0.792 |  | 1.05 (0.74–1.48) | 0.791 |  | 1.05 (0.74–1.48) | 0.793 |
| Quartile 3 | 88 / 1106 | 1.41 (1.02–1.95) | 0.036 |  | 1.42 (1.03–1.96) | 0.034 |  | 1.36 (0.98–1.88) | 0.067 |
| Quartile 4 | 109 / 1120 | 1.76 (1.29–2.40) | <0.001 |  | 1.75 (1.28–2.39) | <0.001 |  | 1.57 (1.15–2.15) | 0.005 |
| Heart disease |  |  |  |  |  |  |  |  |  |
| BMI |  |  |  |  |  |  |  |  |  |
| Quartile 1 | 130 / 1153 | 1 (Reference) |  |  | 1 (Reference) |  |  | 1 (Reference) |  |
| Quartile 2 | 131 / 1103 | 1.05 (0.82–1.34) | 0.708 |  | 1.05 (0.82–1.34) | 0.700 |  | 1.05 (0.82–1.34) | 0.717 |
| Quartile 3 | 138 / 1131 | 1.06 (0.83–1.35) | 0.657 |  | 1.06 (0.83–1.35) | 0.654 |  | 1.01 (0.79–1.30) | 0.909 |
| Quartile 4 | 192 / 1096 | 1.54 (1.22–1.95) | <0.001 |  | 1.55 (1.23–1.96) | <0.001 |  | 1.38 (1.07–1.76) | 0.011 |
| TyG |  |  |  |  |  |  |  |  |  |
| Quartile 1 | 126 / 1121 | 1 (Reference) |  |  | 1 (Reference) |  |  | 1 (Reference) |  |
| Quartile 2 | 143 / 1121 | 1.10 (0.86–1.40) | 0.442 |  | 1.11 (0.87–1.41) | 0.409 |  | 1.07 (0.84–1.36) | 0.586 |
| Quartile 3 | 155 / 1120 | 1.15 (0.91–1.46) | 0.237 |  | 1.15 (0.91–1.46) | 0.239 |  | 1.09 (0.86–1.38) | 0.484 |
| Quartile 4 | 167 / 1121 | 1.29 (1.02–1.62) | 0.034 |  | 1.29 (1.02–1.63) | 0.033 |  | 1.14 (0.89–1.45) | 0.293 |
| hsCRP |  |  |  |  |  |  |  |  |  |
| Quartile 1 | 139 / 1145 | 1 (Reference) |  |  | 1 (Reference) |  |  | 1 (Reference) |  |
| Quartile 2 | 127 / 1112 | 0.96 (0.75–1.22) | 0.710 |  | 0.96 (0.76–1.22) | 0.748 |  | 0.95 (0.74–1.20) | 0.646 |
| Quartile 3 | 165 / 1106 | 1.21 (0.96–1.52) | 0.101 |  | 1.22 (0.97–1.53) | 0.089 |  | 1.17 (0.93–1.46) | 0.188 |
| Quartile 4 | 160 / 1120 | 1.21 (0.96–1.52) | 0.107 |  | 1.21 (0.96–1.52) | 0.101 |  | 1.12 (0.89–1.42) | 0.324 |

Abbreviations: BMI, body mass index; CVD, cardiovascular disease; hsCRP, high-sensitivity C-reactive protein; TyG, triglyceride-glucose index; HR, hazard ratio; CI, confidence interval.

^a^ Adjusted for age, gender, marital status, education level, and residence type.

^b^ Adjusted for age, gender, marital status, education level, residence type, drinking status, smoking status, and night sleep duration.

^c^ Adjusted for age, gender, marital status, education level, residence type, drinking status, smoking status, night sleep duration, depressive symptoms score, hypertension diagnosis, diabetes diagnosis, dyslipidemia diagnosis, kidney disease diagnosis, taking any hypertension medication, taking any diabetes medication, and taking any medication for dyslipidemia.

Table S5. Association between joint trajectories of body mass index, triglyceride-glucose index and high-sensitivity C-reactive protein and incidence of stroke stratified by different factors

| Subgroup | No. of event /  total | Joint trajectories, HR (95% CI) ^a^ | | | | P for  interaction ^a^ |
| --- | --- | --- | --- | --- | --- | --- |
|  |  | LRP | ODP | MOP | IDP |  |
| Age |  |  |  |  |  | 0.589 |
| 45-59 years | 160 / 2680 | 1 (Reference) | 1.37 (0.90–2.07) | 1.61 (1.03–2.52) | 1.91 (1.13–3.24) |  |
| ≥60 years | 165 / 1803 | 1 (Reference) | 1.61 (1.07–2.42) | 1.95 (1.24–3.04) | 1.54 (0.94–2.52) |  |
| Gender |  |  |  |  |  | 0.835 |
| Men | 159 / 2107 | 1 (Reference) | 1.53 (1.00–2.34) | 1.82 (1.14–2.92) | 2.02 (1.27–3.21) |  |
| Women | 166 / 2376 | 1 (Reference) | 1.45 (0.97–2.16) | 1.72 (1.12–2.64) | 1.39 (0.78–2.48) |  |
| Marital status |  |  |  |  |  | 0.257 |
| Married | 273 / 3875 | 1 (Reference) | 1.67 (1.22–2.30) | 1.97 (1.40–2.78) | 1.76 (1.17–2.64) |  |
| Other | 52 / 608 | 1 (Reference) | 0.67 (0.27–1.66) | 1.46 (0.66–3.24) | 1.61 (0.73–3.54) |  |
| Education level |  |  |  |  |  | 0.496 |
| Below high school | 299 / 4084 | 1 (Reference) | 1.57 (1.16–2.13) | 1.87 (1.35–2.59) | 1.78 (1.22–2.58) |  |
| High school | 25 / 361 | 1 (Reference) | 0.86 (0.30–2.50) | 1.44 (0.42–4.95) | 1.83 (0.44–7.71) |  |
| Residence type |  |  |  |  |  | 0.635 |
| Urban | 95 / 1456 | 1 (Reference) | 1.74 (0.97–3.13) | 1.94 (1.06–3.53) | 1.76 (0.82–3.80) |  |
| Rural | 230 / 3027 | 1 (Reference) | 1.42 (1.01–2.00) | 1.84 (1.26–2.69) | 1.81 (1.21–2.72) |  |
| Drinking status |  |  |  |  |  | 0.570 |
| Never | 180 / 2619 | 1 (Reference) | 1.59 (1.08–2.33) | 1.65 (1.08–2.52) | 1.34 (0.79–2.28) |  |
| Former | 33 / 321 | 1 (Reference) | 0.76 (0.28–2.06) | 0.93 (0.31–2.80) | 1.32 (0.46–3.79) |  |
| Current | 112 / 1543 | 1 (Reference) | 1.54 (0.92–2.60) | 2.62 (1.56–4.42) | 2.51 (1.42–4.41) |  |
| Smoking status |  |  |  |  |  | 0.699 |
| Never | 189 / 2751 | 1 (Reference) | 1.57 (1.08–2.28) | 1.75 (1.16–2.65) | 1.43 (0.84–2.45) |  |
| Former | 33 / 326 | 1 (Reference) | 1.56 (0.57–4.29) | 1.24 (0.37–4.11) | 3.60 (1.28–10.17) |  |
| Current | 103 / 1406 | 1 (Reference) | 1.24 (0.70–2.18) | 1.96 (1.14–3.38) | 1.82 (1.03–3.21) |  |
| Hypertension diagnosis |  |  |  |  |  | 0.381 |
| No | 210 / 3563 | 1 (Reference) | 1.56 (1.09–2.24) | 2.17 (1.48–3.18) | 2.13 (1.41–3.22) |  |
| Yes | 115 / 920 | 1 (Reference) | 1.18 (0.70–1.98) | 1.27 (0.73–2.23) | 1.01 (0.50–2.07) |  |
| Diabetes diagnosis |  |  |  |  |  | 0.351 |
| No | 295 / 4254 | 1 (Reference) | 1.52 (1.13–2.04) | 1.69 (1.21–2.36) | 1.73 (1.20–2.50) |  |
| Yes | 30 / 229 | 1 (Reference) | 2.39 (0.42–13.73) | 4.56 (0.98–21.26) | 3.40 (0.45–25.92) |  |
| Dyslipidemia diagnosis |  |  |  |  |  | 0.526 |
| No | 275 / 4155 | 1 (Reference) | 1.52 (1.12–2.08) | 2.00 (1.42–2.81) | 1.86 (1.28–2.71) |  |
| Yes | 50 / 328 | 1 (Reference) | 1.00 (0.42–2.39) | 1.02 (0.43–2.37) | 0.92 (0.27–3.13) |  |
| Kidney disease diagnosis |  |  |  |  |  | 0.546 |
| No | 312 / 4282 | 1 (Reference) | 1.52 (1.13–2.05) | 1.82 (1.32–2.50) | 1.65 (1.14–2.40) |  |
| Yes | 13 / 201 | 1 (Reference) | 2.07 (0.33–12.93) | 3.47 (0.51–23.76) | 8.04 (1.45–44.69) |  |

Abbreviations: IDP, inflammation-dominant profile; LRP, sustained low-risk profile; MOP, metabolic-obesity driven profile; ODP, obesity-predominant profile; HR, hazard ratio; CI, confidence interval.

^a^ Adjusted for age, gender, marital status, education level, residence type, drinking status, smoking status, night sleep duration, depressive symptoms score, hypertension diagnosis, diabetes diagnosis, dyslipidemia diagnosis, kidney disease diagnosis, taking any hypertension medication, taking any diabetes medication, and taking any medication for dyslipidemia, but exclude stratified variable.

Table S6. Association between joint trajectories of body mass index, triglyceride-glucose index and high-sensitivity C-reactive protein and incidence of heart disease stratified by different factors

| Subgroup | No. of event /  total | Joint trajectories, HR (95% CI) ^a^ | | | | P for  interaction ^a^ |
| --- | --- | --- | --- | --- | --- | --- |
|  |  | LRP | ODP | MOP | IDP |  |
| Age |  |  |  |  |  | 0.669 |
| 45-59 years | 305 / 2680 | 1 (Reference) | 1.34 (1.01–1.78) | 1.28 (0.92–1.77) | 1.60 (1.06–2.43) |  |
| ≥60 years | 286 / 1803 | 1 (Reference) | 1.27 (0.94–1.72) | 1.22 (0.86–1.74) | 1.18 (0.80–1.75) |  |
| Gender |  |  |  |  |  | 0.453 |
| Men | 222 / 2107 | 1 (Reference) | 1.47 (1.05–2.08) | 1.27 (0.84–1.93) | 1.11 (0.72–1.73) |  |
| Women | 369 / 2376 | 1 (Reference) | 1.21 (0.94–1.57) | 1.23 (0.92–1.66) | 1.52 (1.05–2.21) |  |
| Marital status |  |  |  |  |  | 0.305 |
| Married | 520 / 3875 | 1 (Reference) | 1.33 (1.07–1.66) | 1.31 (1.01–1.69) | 1.46 (1.08–1.96) |  |
| Other | 71 / 608 | 1 (Reference) | 1.10 (0.59–2.06) | 0.82 (0.38–1.76) | 0.59 (0.22–1.55) |  |
| Education level |  |  |  |  |  | 0.106 |
| Below high school | 537 / 4084 | 1 (Reference) | 1.26 (1.01–1.56) | 1.19 (0.93–1.53) | 1.35 (1.01–1.80) |  |
| High school | 50 / 361 | 1 (Reference) | 2.17 (1.02–4.59) | 2.11 (0.87–5.11) | 1.55 (0.42–5.66) |  |
| Residence type |  |  |  |  |  | 0.629 |
| Urban | 197 / 1456 | 1 (Reference) | 1.37 (0.95–1.98) | 1.47 (1.00–2.18) | 1.26 (0.74–2.16) |  |
| Rural | 394 / 3027 | 1 (Reference) | 1.26 (0.98–1.61) | 1.12 (0.82–1.52) | 1.37 (0.98–1.92) |  |
| Drinking status |  |  |  |  |  | 0.492 |
| Never | 366 / 2619 | 1 (Reference) | 1.19 (0.92–1.55) | 1.15 (0.85–1.56) | 1.43 (1.00–2.06) |  |
| Former | 48 / 321 | 1 (Reference) | 3.00 (1.34–6.69) | 1.79 (0.65–4.95) | 2.73 (1.14–6.55) |  |
| Current | 177 / 1543 | 1 (Reference) | 1.31 (0.90–1.90) | 1.38 (0.90–2.11) | 0.93 (0.52–1.64) |  |
| Smoking status |  |  |  |  |  | 0.143 |
| Never | 406 / 2751 | 1 (Reference) | 1.24 (0.97–1.59) | 1.22 (0.91–1.62) | 1.48 (1.04–2.11) |  |
| Former | 44 / 326 | 1 (Reference) | 1.04 (0.44–2.48) | 1.05 (0.35–3.11) | 1.91 (0.78–4.69) |  |
| Current | 141 / 1406 | 1 (Reference) | 1.63 (1.07–2.50) | 1.41 (0.86–2.31) | 0.88 (0.49–1.60) |  |
| Hypertension diagnosis |  |  |  |  |  | 0.869 |
| No | 421 / 3563 | 1 (Reference) | 1.34 (1.06–1.70) | 1.18 (0.88–1.57) | 1.32 (0.94–1.84) |  |
| Yes | 170 / 920 | 1 (Reference) | 1.18 (0.77–1.81) | 1.31 (0.83–2.08) | 1.32 (0.76–2.30) |  |
| Diabetes diagnosis |  |  |  |  |  | 0.289 |
| No | 543 / 4254 | 1 (Reference) | 1.35 (1.09–1.66) | 1.22 (0.94–1.57) | 1.32 (0.99–1.77) |  |
| Yes | 48 / 229 | 1 (Reference) | 0.60 (0.22–1.65) | 0.99 (0.46–2.14) | 1.12 (0.33–3.76) |  |
| Dyslipidemia diagnosis |  |  |  |  |  | 0.785 |
| No | 511 / 4155 | 1 (Reference) | 1.34 (1.08–1.67) | 1.25 (0.96–1.62) | 1.32 (0.97–1.79) |  |
| Yes | 80 / 328 | 1 (Reference) | 1.02 (0.52–1.99) | 1.14 (0.61–2.16) | 1.51 (0.65–3.54) |  |
| Kidney disease diagnosis |  |  |  |  |  | 0.894 |
| No | 561 / 4282 | 1 (Reference) | 1.27 (1.03–1.57) | 1.22 (0.96–1.56) | 1.31 (0.98–1.76) |  |
| Yes | 30 / 201 | 1 (Reference) | 1.42 (0.53–3.84) | 1.99 (0.59–6.74) | 3.02 (0.76–11.92) |  |

Abbreviations: IDP, inflammation-dominant profile; LRP, sustained low-risk profile; MOP, metabolic-obesity driven profile; ODP, obesity-predominant profile; HR, hazard ratio; CI, confidence interval.

^a^ Adjusted for age, gender, marital status, education level, residence type, drinking status, smoking status, night sleep duration, depressive symptoms score, hypertension diagnosis, diabetes diagnosis, dyslipidemia diagnosis, kidney disease diagnosis, taking any hypertension medication, taking any diabetes medication, and taking any medication for dyslipidemia, but exclude stratified variable.

Table S7. Association between joint trajectories of body mass index, triglyceride-glucose index and high-sensitivity C-reactive protein and incidence of cardiovascular diseases in subpopulations of 4,133 participants with complete data

| Outcomes | No. of event /  total | Model 1 ^a^ | |  | Model 2 ^b^ | |  | Model 3 ^c^ | |
| --- | --- | --- | --- | --- | --- | --- | --- | --- | --- |
|  |  | HR (95% CI) | P value |  | HR (95% CI) | P value |  | HR (95% CI) | P value |
| CVD |  |  |  |  |  |  |  |  |  |
| LRP | 286 / 1916 | 1 (Reference) |  |  | 1 (Reference) |  |  | 1 (Reference) |  |
| ODP | 229 / 1112 | 1.44 (1.21–1.73) | <0.001 |  | 1.45 (1.21–1.74) | <0.001 |  | 1.35 (1.12–1.62) | 0.001 |
| MOP | 166 / 715 | 1.68 (1.38–2.04) | <0.001 |  | 1.69 (1.39–2.06) | <0.001 |  | 1.44 (1.17–1.77) | <0.001 |
| IDP | 93 / 390 | 1.66 (1.31–2.10) | <0.001 |  | 1.65 (1.30–2.09) | <0.001 |  | 1.55 (1.22–1.96) | <0.001 |
| Stroke |  |  |  |  |  |  |  |  |  |
| LRP | 101 / 1916 | 1 (Reference) |  |  | 1 (Reference) |  |  | 1 (Reference) |  |
| ODP | 83 / 1112 | 1.60 (1.19–2.16) | 0.002 |  | 1.60 (1.19–2.16) | 0.002 |  | 1.42 (1.04–1.93) | 0.025 |
| MOP | 75 / 715 | 2.31 (1.70–3.13) | <0.001 |  | 2.32 (1.71–3.14) | <0.001 |  | 1.80 (1.30–2.49) | <0.001 |
| IDP | 42 / 390 | 2.05 (1.43–2.94) | <0.001 |  | 2.03 (1.41–2.91) | <0.001 |  | 1.85 (1.28–2.65) | 0.001 |
| Heart disease |  |  |  |  |  |  |  |  |  |
| LRP | 207 / 1916 | 1 (Reference) |  |  | 1 (Reference) |  |  | 1 (Reference) |  |
| ODP | 167 / 1112 | 1.38 (1.12–1.70) | 0.003 |  | 1.38 (1.12–1.71) | 0.002 |  | 1.31 (1.05–1.62) | 0.015 |
| MOP | 108 / 715 | 1.43 (1.13–1.82) | 0.003 |  | 1.45 (1.14–1.83) | 0.002 |  | 1.27 (0.99–1.63) | 0.055 |
| IDP | 60 / 390 | 1.48 (1.11–1.98) | 0.007 |  | 1.48 (1.11–1.97) | 0.008 |  | 1.40 (1.05–1.87) | 0.023 |

Abbreviations: CVD, cardiovascular diseases; IDP, inflammation-dominant profile; LRP, sustained low-risk profile; MOP, metabolic-obesity driven profile; ODP, obesity-predominant profile; HR, hazard ratio; CI, confidence interval.

^a^ Adjusted for age, gender, marital status, education level, and residence type.

^b^ Adjusted for age, gender, marital status, education level, residence type, drinking status, smoking status, and night sleep duration.

^c^ Adjusted for age, gender, marital status, education level, residence type, drinking status, smoking status, night sleep duration, depressive symptoms score, hypertension diagnosis, diabetes diagnosis, dyslipidemia diagnosis, kidney disease diagnosis, taking any hypertension medication, taking any diabetes medication, and taking any medication for dyslipidemia.

Table S8. Association between joint trajectories of body mass index, triglyceride-glucose index and high-sensitivity C-reactive protein and incidence of cardiovascular diseases with further adjustment for additional covariates.

| Outcomes | No. of event /  total | Model 3 ^a^ | |  | Model 4 ^b^ | |
| --- | --- | --- | --- | --- | --- | --- |
|  |  | HR (95% CI) | P value | T | HR (95% CI) | P value |
| CVD |  |  |  |  |  |  |
| LRP | 316 / 2100 | 1 (Reference) |  |  | 1 (Reference) |  |
| ODP | 252 / 1195 | 1.35 (1.13–1.60) | 0.001 |  | 1.25 (1.05–1.50) | 0.014 |
| MOP | 176 / 767 | 1.41 (1.15–1.71) | 0.001 |  | 1.27 (1.02–1.58) | 0.032 |
| IDP | 95 / 421 | 1.46 (1.16–1.84) | 0.001 |  | 1.36 (1.07–1.72) | 0.012 |
| Stroke |  |  |  |  |  |  |
| LRP | 110 / 2100 | 1 (Reference) |  |  | 1 (Reference) |  |
| ODP | 94 / 1195 | 1.50 (1.12–2.01) | 0.006 |  | 1.36 (1.01–1.83) | 0.045 |
| MOP | 79 / 767 | 1.83 (1.34–2.50) | <0.001 |  | 1.57 (1.11–2.22) | 0.011 |
| IDP | 42 / 421 | 1.75 (1.22–2.50) | 0.002 |  | 1.54 (1.06–2.22) | 0.022 |
| Heart disease |  |  |  |  |  |  |
| LRP | 230 / 2100 | 1 (Reference) |  |  | 1 (Reference) |  |
| ODP | 183 / 1195 | 1.29 (1.05–1.59) | 0.015 |  | 1.22 (0.99–1.51) | 0.060 |
| MOP | 116 / 767 | 1.25 (0.98–1.58) | 0.070 |  | 1.14 (0.88–1.49) | 0.318 |
| IDP | 62 / 421 | 1.33 (1.00–1.76) | 0.048 |  | 1.26 (0.95–1.68) | 0.114 |

Abbreviations: HR, hazard ratio; CI, confidence interval.

^a^ Adjusted for age, gender, marital status, education level, residence type, drinking status, smoking status, night sleep duration, depressive symptoms score, hypertension diagnosis, diabetes diagnosis, dyslipidemia diagnosis, kidney disease diagnosis, taking any hypertension medication, taking any diabetes medication, and taking any medication for dyslipidemia.

^b^ Adjusted for age, gender, marital status, education level, residence type, drinking status, smoking status, night sleep duration, depressive symptoms score, hypertension diagnosis, diabetes diagnosis, dyslipidemia diagnosis, kidney disease diagnosis, taking any hypertension medication, taking any diabetes medication, taking any medication for dyslipidemia, systolic blood pressure, diastolic blood pressure, high-density lipoprotein, low-density lipoprotein, estimated glomerular filtration rate, and glycated hemoglobin.

Table S9. Association between joint trajectories of body mass index, triglyceride-glucose index and high-sensitivity C-reactive protein and incidence of cardiovascular diseases excluding participants with incident cardiovascular disease during the two year of follow-up

| Outcomes | No. of event /  total | Model 1 ^a^ | |  | Model 2 ^b^ | |  | Model 3 ^c^ | |
| --- | --- | --- | --- | --- | --- | --- | --- | --- | --- |
|  |  | HR (95% CI) | P value |  | HR (95% CI) | P value |  | HR (95% CI) | P value |
| CVD |  |  |  |  |  |  |  |  |  |
| LRP | 87 / 1712 | 1 (Reference) |  |  | 1 (Reference) |  |  | 1 (Reference) |  |
| ODP | 76 / 959 | 1.60 (1.17–2.19) | 0.004 |  | 1.64 (1.19–2.26) | 0.002 |  | 1.59 (1.14–2.20) | 0.006 |
| MOP | 60 / 609 | 2.26 (1.62–3.17) | <0.001 |  | 2.32 (1.66–3.26) | <0.001 |  | 2.07 (1.45–2.96) | <0.001 |
| IDP | 26 / 304 | 1.74 (1.12–2.70) | 0.013 |  | 1.73 (1.11–2.68) | 0.015 |  | 1.66 (1.07–2.58) | 0.025 |
| Stroke |  |  |  |  |  |  |  |  |  |
| LRP | 19 / 1712 | 1 (Reference) |  |  | 1 (Reference) |  |  | 1 (Reference) |  |
| ODP | 22 / 959 | 2.20 (1.18–4.12) | 0.013 |  | 2.34 (1.25–4.39) | 0.008 |  | 2.31 (1.21–4.39) | 0.011 |
| MOP | 25 / 609 | 4.39 (2.39–8.08) | <0.001 |  | 4.57 (2.47–8.43) | <0.001 |  | 4.02 (2.11–7.67) | <0.001 |
| IDP | 12 / 304 | 3.61 (1.75–7.44) | 0.001 |  | 3.57 (1.73–7.37) | 0.001 |  | 3.44 (1.66–7.14) | 0.001 |
| Heart disease |  |  |  |  |  |  |  |  |  |
| LRP | 72 / 1712 | 1 (Reference) |  |  | 1 (Reference) |  |  | 1 (Reference) |  |
| ODP | 56 / 959 | 1.40 (0.98–2.01) | 0.066 |  | 1.42 (0.99–2.04) | 0.057 |  | 1.36 (0.93–1.97) | 0.109 |
| MOP | 39 / 609 | 1.77 (1.19–2.64) | 0.005 |  | 1.81 (1.22–2.70) | 0.004 |  | 1.62 (1.06–2.46) | 0.025 |
| IDP | 17 / 304 | 1.39 (0.82–2.37) | 0.219 |  | 1.38 (0.81–2.34) | 0.236 |  | 1.32 (0.77–2.24) | 0.313 |

Abbreviations: CVD, cardiovascular diseases; IDP, inflammation-dominant profile; LRP, sustained low-risk profile; MOP, metabolic-obesity driven profile; ODP, obesity-predominant profile; HR, hazard ratio; CI, confidence interval.

^a^ Adjusted for age, gender, marital status, education level, and residence type.

^b^ Adjusted for age, gender, marital status, education level, residence type, drinking status, smoking status, and night sleep duration.

^c^ Adjusted for age, gender, marital status, education level, residence type, drinking status, smoking status, night sleep duration, depressive symptoms score, hypertension diagnosis, diabetes diagnosis, dyslipidemia diagnosis, kidney disease diagnosis, taking any hypertension medication, taking any diabetes medication, and taking any medication for dyslipidemia.

**Table S10. Calinski & Harabasz criterion**

| Number of trajectories | Calinski value ^a^ | Harabasz value ^a^ |
| --- | --- | --- |
| 2 | 1214.947 | 1213.909 |
| 3 | 1219.891 | 1218.178 |
| 4 | 1236.503 | 1234.619 |
| 5 | 1107.787 | 1106.545 |

^a^ High value denoting good partition.

**Table S11. Association between joint trajectories of body mass index, triglyceride-glucose index and high-sensitivity C-reactive protein and incidence of cardiovascular diseases after random exclusion of 20% of participants**

| Outcomes | No. of event /  total | Model 1 ^a^ | |  | Model 2 ^b^ | |  | Model 3 ^c^ | |
| --- | --- | --- | --- | --- | --- | --- | --- | --- | --- |
|  |  | HR (95% CI) | P value |  | HR (95% CI) | P value |  | HR (95% CI) | P value |
| CVD |  |  |  |  |  |  |  |  |  |
| LRP | 248 / 1678 | 1 (Reference) |  |  | 1 (Reference) |  |  | 1 (Reference) |  |
| ODP | 204 / 973 | 1.50 (1.24–1.82) | <0.001 |  | 1.52 (1.25–1.83) | <0.001 |  | 1.40 (1.15–1.70) | 0.001 |
| MOP | 141 / 600 | 1.75 (1.42–2.16) | <0.001 |  | 1.77 (1.43–2.18) | <0.001 |  | 1.52 (1.22–1.90) | <0.001 |
| IDP | 66 / 335 | 1.40 (1.07–1.84) | 0.016 |  | 1.40 (1.07–1.84) | 0.014 |  | 1.32 (1.00–1.73) | 0.047 |
| Stroke |  |  |  |  |  |  |  |  |  |
| LRP | 87 / 1678 | 1 (Reference) |  |  | 1 (Reference) |  |  | 1 (Reference) |  |
| ODP | 69 / 973 | 1.54 (1.11–2.12) | 0.009 |  | 1.55 (1.12–2.15) | 0.008 |  | 1.35 (0.97–1.88) | 0.079 |
| MOP | 64 / 600 | 2.39 (1.72–3.32) | <0.001 |  | 2.41 (1.74–3.35) | <0.001 |  | 1.91 (1.34–2.70) | <0.001 |
| IDP | 31 / 335 | 1.81 (1.20–2.72) | 0.005 |  | 1.81 (1.20–2.74) | 0.004 |  | 1.64 (1.08–2.48) | 0.019 |
| Heart disease |  |  |  |  |  |  |  |  |  |
| LRP | 178 / 1678 | 1 (Reference) |  |  | 1 (Reference) |  |  | 1 (Reference) |  |
| ODP | 157 / 973 | 1.55 (1.25–1.94) | <0.001 |  | 1.56 (1.25–1.95) | <0.001 |  | 1.48 (1.18–1.85) | 0.001 |
| MOP | 94 / 600 | 1.57 (1.22–2.02) | <0.001 |  | 1.58 (1.22–2.03) | <0.001 |  | 1.41 (1.08–1.84) | 0.011 |
| IDP | 41 / 335 | 1.21 (0.86–1.70) | 0.271 |  | 1.22 (0.87–1.72) | 0.246 |  | 1.17 (0.83–1.65) | 0.355 |

Abbreviations: CVD, cardiovascular diseases; IDP, inflammation-dominant profile; LRP, sustained low-risk profile; MOP, metabolic-obesity driven profile; ODP, obesity-predominant profile; HR, hazard ratio; CI, confidence interval.

^a^ Adjusted for age, gender, marital status, education level, and residence type.

^b^ Adjusted for age, gender, marital status, education level, residence type, drinking status, smoking status, and night sleep duration.

^c^ Adjusted for age, gender, marital status, education level, residence type, drinking status, smoking status, night sleep duration, depressive symptoms score, hypertension diagnosis, diabetes diagnosis, dyslipidemia diagnosis, kidney disease diagnosis, taking any hypertension medication, taking any diabetes medication, and taking any medication for dyslipidemia.
